# Endoplasmic reticulum-mitochondria crosstalk: new mechanisms in the development of atherosclerosis

**DOI:** 10.3389/fendo.2025.1573499

**Published:** 2025-06-05

**Authors:** Mingxiao Li, Yili Xiao, Ling Dai, Simin Chen, Wanjuan Pei, Chao Tan

**Affiliations:** ^1^ Medical School, Hunan University of Chinese Medicine, Changsha, China; ^2^ Department of Cardiology, The First Affiliated Hospital of Hunan University of Chinese Medicine, Changsha, China; ^3^ The Domestic First-class Discipline Construction Project of Chinese Medicine of Hunan University of Chinese Medicine, Changsha, China

**Keywords:** atherosclerosis, endoplasmic reticulum-mitochondrial crosstalk, mitochondria-associated membranes (MAMs), endoplasmic reticulum contact complex, endoplasmic reticulum, mitochondria

## Abstract

Atherosclerosis (AS) is a global public health concern and involves a complex pathogenesis characterized by lipid abnormalities, oxidative stress, and inflammatory responses at the cellular and molecular levels. The crosstalk between the endoplasmic reticulum (ER) and mitochondria, mediated by mitochondria-associated membranes (MAMs), plays a critical role in the pathogenesis of atherosclerosis. As two key cellular organelles, the ER and mitochondria interact physically and functionally through MAMs, which serve as bridges between their close contact and interdependence. MAMs maintain lipid homeostasis, promote calcium ion transport, the oxidative stress response, apoptosis, and autophagy. Recent studies have highlighted the significance of ER-mitochondria crosstalk in the progression of AS, as indicated by mitochondrial and ER structural and functional integrity, redox homeostasis, and calcium homeostasis. This review comprehensively explores the novel mechanisms of ER-mitochondria crosstalk in AS and emphasizes the potential of MAMs as therapeutic targets, aiming to provide new perspectives and strategies for the treatment of cardiovascular diseases.

## 1 Introduction

Atherosclerosis (AS), characterized by lipid accumulation, chronic inflammation, and vascular dysfunction, is a complex pathological process and a core contributor to global cardiovascular disease-related disability and mortality (1). With improvements in living standards and dietary changes, its incidence has increased annually, severely threatening human quality of life (2). According to the 2019 Global Burden of Disease (GBD) statistics, approximately one-third of annual deaths are attributed to cardiovascular diseases, with AS-related complications being the dominant factor (3). Although statins, anti-inflammatory therapies, and vascular intervention technologies have significantly improved clinical outcomes, acute cardiovascular events (e.g., myocardial infarction and stroke) caused by plaque instability remain a major challenge (4). Therefore, there is an urgent need to explore the molecular mechanisms underlying AS.

Traditionally, AS research has focused on singular pathological processes such as the oxidation of low-density lipoprotein (LDL), macrophage foam cell transformation, and smooth muscle cell proliferation; however, the dynamic interactions between organelles that systemically facilitate disease progression have been overlooked. Recent breakthroughs in super-resolution microscopy, spatial transcriptomics, and protein interactionomics have propelled the study of organelle communication networks to the forefront of life sciences (5). Notably, the physical and functional coupling between the endoplasmic reticulum (ER) and mitochondria via mitochondria-associated membranes (MAMs) has been identified as a core hub for coordinating lipid metabolism, calcium signaling, oxidative stress, and cell fate decisions (6–9), offering a novel perspective for AS research.

The ER, the largest membrane system in the cell, performs fundamental functions such as protein synthesis and modification, lipid generation, and calcium storage, while also serving as a platform for stress sensing and signal integration (10). Mitochondria drive energy metabolism and inflammatory responses via oxidative phosphorylation and reactive oxygen species (ROS) generation (11). Over the past decade, research has revealed that the ER and mitochondria are not independent but are functionally coupled through MAMs. MAMs are dynamic structures formed by specific protein anchors between the ER and mitochondrial membranes, enabling the efficient transport of lipids, calcium ions, and signaling molecules (12). For example, calcium flux mediated by the IP3R-GRP75-voltage-dependent anion channel (VDAC) complex (13), mitochondrial autophagy promoted by FUN14 domain-containing protein 1 (FUNDC1) (6), and membrane fusion events involving Mitofusin 2 (Mfn2) (7) depend on the precise spatiotemporal regulation of MAMs.

AS is a vascular pathology driven by metabolic and inflammatory synergies. Pathological conditions, such as endothelial cell damage induced by oxidized LDL (ox-LDL), macrophage inflammatory polarization, and smooth muscle cell phenotype switching, are accompanied by ER stress and mitochondrial dysfunction (14, 15). Recent studies have identified multiple mechanisms by which MAMs contribute to these processes: (1) lipid metabolism, where MAM-enriched enzymes such as ACAT1 and phosphatidylethanolamine N-methyltransferase 2 (PEMT2) facilitate cholesterol esterification and phospholipid remodeling, promoting lipid droplet formation and foam cell transformation (16); (2) inflammation regulation, where MAMs serve as a platform for NLRP3 inflammasome assembly, amplifying inflammatory signals through calcium overload and mitochondrial reactive oxygen species (mtROS) bursts (17); (3) apoptosis, where MAMs promote interactions between Bcl-2 family proteins and mitochondrial membrane permeability transitions, determining cellular survival within plaques (18).

Despite significant progress, many gaps remain in our understanding of the molecular mechanisms of MAMs in AS. For instance, how do MAMs dynamically assemble in response to mechanical stimuli such as blood flow shear forces? Do specific cell types (e.g., endothelial cells and macrophages) exhibit spatial heterogeneity in MAM function? Are there temporal heterogeneities in the MAMs function across different disease phases? Existing drugs (e.g., statins (19) and metformin (20)) have been shown to modulate ER-mitochondrial crosstalk by targeting key MAM proteins (e.g., VDAC1 and Mfn2); however, their multi-target characteristics may lead to off-target effects, highlighting the need for developing specific MAM-targeted therapies. Addressing these questions requires the integration of multi-omics analysis, organoid models, and gene editing technologies to further dissect the regulatory network of MAMs across spatiotemporal dimensions and provide a comprehensive strategy for AS treatment.

This review aims to systematically summarize the latest research progress on ER-mitochondria crosstalk in AS, focusing on the structural and functional characteristics of MAMs and their regulatory roles in lipid metabolism, inflammatory responses, and cell death. By organizing key molecular mechanisms and evaluating their clinical translation potential, we aim to reveal innovative therapeutic strategies targeting MAMs and provide a theoretical framework for the development of precision medicine based on organelle interaction regulation.

## 2 The structural basis of ER-mitochondria crosstalk

The signaling network between the ER and mitochondria constitutes a complex and precise system within the cell. Research on physical and functional interactions between these organelles has primarily focused on physical connections (e.g., MAMs and Endoplasmic Reticulum-Mitochondria Encounter Structure [ERMES]), which provide direct channels for material exchange (21–24). ERMES also contributes to maintaining the mitochondrial function (25–29). Additionally, proteins on MAMs and ERMES participate in various signaling pathways, thereby promoting calcium signaling (22–24), cellular stress and apoptosis (30–35), and other processes. These interactions not only facilitate material exchange and energy metabolism coordination but also provide essential protective mechanisms for cells to respond to environmental changes.

### 2.1 mitochondria-associated membranes

MAMs, which are the membrane structures between mitochondria and the ER, were first described by Copeland et al. (12) and confirmed in subsequent studies. Despite the narrow membrane gap of MAMs (10–80 nm), these structures efficiently support multiple critical biological processes including calcium homeostasis, lipid metabolism, autophagy, inflammatory responses, ER stress, and mitochondrial dynamics (36).

MAMs enable a more direct material exchange and information communication between the ER and mitochondria, a process that relies on diverse proteins within MAMs. Proteins in MAMs are categorized into three types: (1) Proteins exclusively localized to MAMs (MAMs-resident proteins), (2) Proteins localized to MAMs but also present in other cellular regions (MAMs-enriched proteins), (3) Proteins transiently associated with MAMs (MAMs-associated proteins) (37). Owing to the dynamic nature of MAMs, their exact composition remains unclear.

MAMs contain numerous proteins that perform diverse functions and regulate various cellular and biological processes. Enriched proteins in MAMs, such as glucose-regulated protein 75 (GRP75) and inositol 1,4,5-trisphosphate receptor (IP3R), contribute to maintaining the structure and function of MAMs. IP3R participates in ER calcium release. FUNDC1 typically acts as a mitochondrial autophagy receptor, while Sigma-1 receptor (Sig-1R) regulates ER stress, mitochondrial function, and oxidative stress (6). Mfn2, an important MAM-enriched protein, maintains calcium homeostasis, and mitochondrial dynamics. Mfn2 is believed to protect mitochondria and inhibit apoptosis by suppressing activation of the PERK pathway (7). Increased Mfn2 expression can also ameliorate mitochondrial calcium overload (8). MAM-resident proteins, such as phosphatidylserine synthase 1/2 (PSS1/2), are highly enriched in MAMs and participate in the transport of phospholipids between the mitochondria and ER, thereby promoting lipid synthesis. Mitofusin 1 (Mfn1) collaborates with other MAM-enriched proteins to maintain the structure of MAMs (6, 9).

As a key site for ER-mitochondrial crosstalk, MAMs, with their intricate structure, participate in and regulate various physiological and pathological processes, and play a crucial role in maintaining cellular homeostasis and responding to diverse stress reactions. Related studies are ongoing.

### 2.2 Endoplasmic reticulum-mitochondria encounter structure

The endoplasmic reticulum-mitochondrial encounter structure (ERMES) acts as a bridge and regulatory center within the cell. ERMES is a multi-subunit complex composed of transmembrane anchoring components (ER membrane protein Mmm1 and mitochondrial outer membrane proteins Mdm10 and Mdm34), soluble connecting components (Mdm12 interacts with the synaptic binding protein-like mitochondrial lipid-binding protein [SMP] domain of Mmm1 and Mdm34 to stabilize ERMES conformation (11)), and dynamic regulatory components (RhoGTPase Gem1 hydrolyzes GTP to assemble and disassemble ERMES, though its presence is condition-dependent and not all ERMES complexes contain this subunit (10)). The SMP domains of Mdm34, Mdm12, and Mmm1 specifically interact to form a stable complex that bridges the ER and mitochondrial outer membranes. Two Mdm12 and two Mmm1 SMP domains interact in a head-to-tail manner to form a tetrameric hydrophobic channel (12), providing structural support for transmembrane transport (e.g., Ca^2+^, phospholipids) and signal communication.

Gem1, Mdm10, and Mmm1 play key roles in maintaining mitochondrial morphology. Gem1 has two Ca^2+^-binding EF-hand motifs, and Ca^2+^ from the ER can bind to these motifs to activate Gem1, promoting Ca^2+^ transfer to mitochondria and thereby regulating mitochondrial movement (13). Therefore, the ER can influence mitochondrial morphology through the ERMES (38). Rasul et al. (14) demonstrated that Mdm12 interacts with the MAMs regulatory protein Emr1, and that in the absence of Emr1, the number of ERMES structures decreases, leading to abnormalities in mitochondrial morphological. This study further confirms that ERMES mediates mitochondrial dynamics.

Various cellular processes mediated by ERMES are influenced by ERMES regulatory proteins. In addition to Emr1, Arf1, Lam6, and Gem1 help to maintain the number of ERMES foci. Overexpression of Lam6 causes ERMES expansion, Tom7 increases the specificity of Mdm10 binding to ERMES, preventing excessive leakage of Mdm10 from ERMES and binding to the SAM complex, and Sar1 regulates the area of the ERMES complex (15).

## 3 Biological processes of endoplasmic reticulum-mitochondria crosstalk

### 3.1 Lipid synthesis

Lipid synthesis is not confined to the ER; multiple enzymes in MAMs participate in this process. For example, in the synthesis of phosphatidylcholine (PC) and phosphatidylethanolamine (PE), phosphatidylserine synthase 1 (PSS1) in the ER catalyzes the formation of phosphatidylserine (PS) from phosphatidic acid (PA). PS enters the mitochondria via MAMs and is converted to PE by related enzymes. PE is then transported out of the mitochondria and converted to PC by PEMT2 in the ER (17). PEMT2, a key enzyme in PC synthesis, has only been identified in MAMs (18). Studies have explored the mechanism of PS entry into the mitochondria. In the liver tissue of mice with non-alcoholic steatohepatitis (NASH), Mfn2 expression is significantly reduced. Mfn2 knockout in mouse hepatocytes causes triglyceride accumulation and inflammatory responses (19). Hernández et al. demonstrated that Mfn2 binds to PS and transports it to the mitochondria for subsequent reactions (19). Enzymes involved in phospholipid biosynthesis are present in both ER and mitochondrial membranes, and the intermediates generated during this process are translocated to ER-mitochondria contact sites (20).

The rate-limiting enzyme in cholesterol synthesis, 3-hydroxy-3-methylglutaryl-coenzyme A reductase (HMGCR), is inhibited by statins, which are commonly used to treat AS. Godoy et al. (39) found that mice treated with atorvastatin lacked ER-mitochondria connections, but this was not proven to be based on HMGCR inhibition. Zhong et al. (40) experimentally confirmed that in aldehyde dehydrogenase 2 (ALDH2)-deficient mice, serum total cholesterol and HMGCR levels increased due to ALDH2 promotion of HMGCR ubiquitination and degradation. This process relies on ALDH2 escaping mitochondria to promote the binding of Insig1 to HMGCR in the ER and recruit GP78, indicating that ER-mitochondrial crosstalk is involved in HMGCR-regulated cholesterol synthesis.

Acyl-CoA:diacylglycerol acyltransferase 2 (DGAT2) in MAMs catalyzes the synthesis, and promotes the formation, of lipid droplets. Acyl-CoA:cholesterol acyltransferase 1 (ACAT1/SOAT1), a key rate-limiting enzyme in MAMs, catalyzes the esterification of free cholesterol with long-chain fatty acids to form cholesterol esters (CE), thereby driving CE storage within lipid droplets and directly regulating the early stages of lipid droplet biogenesis (21). Presenilin 2 (PS2), a protein highly enriched in MAMs, corrects excessive lipid droplet formation in Alzheimer’s disease (AD) cells (22). Zhao et al. (23) found that the ATAD3A protein is enriched in MAMs of AD mouse brain tissue and that ATAD3A oligomerization inhibits CYP46A1-mediated brain cholesterol metabolism in AD mice. Szabo et al. (24) demonstrated that abnormal TAU disrupts ER-mitochondria coupling, reduces cholesterol transfer from the ER to the mitochondria, and decreases pregnenolone production, highlighting the importance of ER-mitochondria crosstalk in lipid synthesis. Phosphofurin acidic cluster sorting protein 2 (PACS2), an early MAM protein, regulates lipid homeostasis. Arruda et al. (25) found that PACS2 and IP3R1/2 levels are significantly increased in tissues from overweight mice. Reducing the expression of PACS2 and IP3R1/2 improves mitochondrial antioxidant capacity and insulin sensitivity in obese animals. In summary, collaboration between the ER and mitochondria ensures the smooth progression of lipid synthesis, thereby maintaining cellular membrane integrity and normal cellular function.

### 3.2 Inflammasome formation

Inflammasomes are signaling complexes that play crucial roles in immune and inflammatory responses. Among the various family members, the most well characterized are NLRP3, NLRP1, NLRC4, and AIM2 (26). ER-mitochondria crosstalk (MAMs) regulates activation of the NLRP3 inflammasome. ER stress induces mitochondrial damage, leading to the release of mtROS and mitochondrial damage-associated molecular patterns (mtDAMPs) such as mitochondrial DNA (mtDNA) and cardiolipin. These are transmitted via MAMs to NLRP3 molecules on the ER membrane, triggering oligomerization and inflammasome activation (27, 28). Moreover, overexpression of RTN1A, a key MAMs protein, can disrupt the binding of HK1 to VDAC1, leading to free VDAC1 driving inflammasome assembly (29). extracellular ATP, through the P2X7R-mediated pathway, disrupts the functional balance of MAMs and induces the assembly of the NLRP3 inflammasome. GRP75 can inhibit eATP-P2X7R pathway-induced NLRP3 inflammasome aggregation (30).

The NLRP3 inflammasome is the only inflammasome currently confirmed to be associated with MAMs and exerts unique functions by sensing damage-associated molecular patterns (DAMPs) in the MAMs microenvironment. DAMPs (e.g., Ca^2+^ signaling, mtROS) can be produced by damaged cells, with Ca^2+^ signaling activating the NLRP3 inflammasome through calcium flux between the ER and mitochondria (31). Ye et al. (32) further investigated the relationship between Ca^2+^ and inflammation and finding that IP3R-mediated excessive Ca^2+^ release could induce mitochondrial dysfunction and promote NLRP3 inflammasome activation. Based on the above studies, it is evident that ER-mitochondrial crosstalk can activate NLRP3. However, inhibition of NLRP3 is also related to MAMs. Missiroli et al. found that PML, located in MAMs, inhibits NLRP3 activation by tightly binding to NLRP3 and P2X7R (33).

### 3.3 Calcium flux and signal transduction

One of the key roles of MAMs is to maintain calcium homeostasis between the ER and mitochondria. Arruda et al. (25) demonstrated that an increase in MAMs under obese conditions exacerbates mitochondrial calcium accumulation and disrupts mitochondrial function. Moreover, when subjected to external stimuli, changes in various components of MAMs can lead to MAMs dysfunction, thereby disrupting Ca^2+^ transport between the ER and mitochondria (36).

MAMs regulates calcium ion flux through several key proteins. FUNDC1, by binding to IP3R2 and localizing to MAMs, increases mitochondrial and cytosolic Ca^2+^ levels while decreasing ER Ca^2+^ levels when overexpressed. Conversely, in the absence of FUNDC1, mitochondrial and cytosolic Ca^2+^ levels decreased, while ER Ca^2+^ levels increase (37). In addition, the IP3R-GRP75-VDAC complex is a classic structure in MAMs involved in calcium homeostasis, facilitating Ca^2+^ transfer from the ER to the mitochondria (41). IP3R mediates Ca^2+^ release from the ER, whereas VDAC is responsible for Ca^2+^ transfer into the mitochondrial intermembrane space. PTEN and Akt, which are localized in MAMs, promote ER calcium release by phosphorylating IP3R. PTEN knockout inhibits ER Ca^2+^ release, thereby maintaining mitochondrial Ca^2+^ homeostasis and negatively regulating apoptosis (42, 43). The Sig-1R-BiP complex in MAMs can rapidly respond to decreases in Ca^2+^ concentration, thereby regulating ER-mitochondria Ca^2+^ signaling (44). Additionally, SERCA2b, enriched in MAMs, exhibits high Ca^2+^ affinity. Its activity is enhanced through interaction with CANX (calnexin) and inhibited via interaction with TMX1 (thioredoxin-related transmembrane protein 1), thereby regulating Ca^2+^ influx into either the endoplasmic reticulum (ER) or mitochondria. (45). Beyond ER Ca^2+^ release and ER-mitochondrial Ca^2+^ transfer, MAM-associated proteins also inhibit mitochondrial Ca^2+^ release. For example, Bcl-2 can reduce mitochondrial calcium release, thereby inhibiting apoptosis (38).

### 3.4 Mitochondrial dynamics and homeostasis

Mitochondria undergo dynamic changes in shape, size, and number via fusion and fission. Previous studies have hypothesized that ER plays a significant role in important mitochondrial functions, including mitochondrial dynamics (46). Under the action of certain mitochondrial dynamic proteins, including MFF, dynamin‐related protein 1(Drp1) accumulates on the mitochondrial outer membrane and is arranged in a helical pattern, causing mitochondrial fission (47). However, in the experiments by Friedman et al. (48), MAMs exhibited abnormal mitochondrial contractions in the absence of MFF. Arasaki et al. (49) further elaborated on this view, indicating that the SNARE protein Syn17 on MAMs activates Drp1 and determines its localization, thereby promoting mitochondrial fission. Under conditions of energy stress, AMPK accumulates extensively and interacts with Mfn2 in MAMs, promoting mitochondrial fission (50). Under hypoxic conditions, FUNDC1 accumulates in MAMs and binds to Drp1, promoting Drp1-mediated mitochondrial fission. Inhibition of FUNDC1 disrupts MAMs integrity, leading to decreased cytoplasmic and mitochondrial Ca^2+^ concentrations and the suppression of mitochondrial fission (24, 51).

The ERMES is often associated with mitochondrial dynamics. During cell division, Mmm1, Mdm10, and Mdm12 connect mitochondria to the actin cytoskeleton and participate in polarized mitochondrial movement (38). ERMES plays a widespread role in the regulation of mitochondrial homeostasis in fungi. Garrido-Bazán et al. (39) found that downregulation of MdmB expression in yeast ERMES leads to mitochondrial fission defects. Experiments also showed that ER-mitochondrial crosstalk is indispensable for H_2_O_2_-induced mitochondrial contraction. In an earlier study, Sogo et al. (40) observed changes in mitochondrial networks and aggregation in yeast Mdm10 mutants. Similarly, giant spherical mitochondria have been discovered after Mdm12 knockout in cells (52). Regarding the specific mechanism, Esposito et al. (53) demonstrated that the absence of Mdm10 or Mdm12 in ERMES leads to an increase in peroxisomes, causing mitochondrial dysfunction. Their experiments provide further evidence for the role of ERMES in maintaining mitochondrial homeostasis.

### 3.5 Autophagy and apoptosis

Autophagy, a universal cellular metabolic process, is characterized by the formation of autophagosomes. Hamasaki et al. found that the autophagy-related protein ATG14 is located in MAMs, where autophagosome formation begins (54). During mitophagy induction, PINK1 and Beclin 1 are expressed in MAMs, promoting the formation of autophagosomes and MAMs (55). The autophagy-related proteins Atg8 and Atg11 co-localize with Mdm12 and Mdm34 in ERMES, facilitating mitophagy (56).

ER-mitochondrial crosstalk provides the structural basis for autophagy. Kohle et al. (28) suggested that the contact between the ER and mitochondria is central to autophagy. In cases of myocardial ischemia-reperfusion injury, Mfn2 can promote the transfer of phospholipids from the ER to the mitochondria, facilitating autophagosome membrane formation, and thereby activating protective mitophagy processes (25). Ikeda et al. (26) experimentally demonstrated that downregulation of Drp1 expression in mouse cells leads to mitochondrial dysfunction, indicating a positive correlation between Drp1 expression and the intensity of Mitophagy. Additionally, the STX17-Fis axis is involved in inducing mitochondrial autophagy; Fis1 inhibits the transfer of STX17 from MAMs to the mitochondria, thereby suppressing mitochondrial phagocytosis during autophagy (27). In summary, ER-mitochondria contact determines mitochondrial fission points, which are a prerequisite for subsequent engulfment by autophagosomes or lysosomes. Böckler et al. (29) further confirmed that ERMES co-localizes with phagophore sites and promotes phagophore membrane formation, possibly by facilitating lipid transfer to phagophores. Böckler et al. suggested that ERMES functions only in mitochondrial autophagy and not in the broader autophagy processes (57). Garofalo et al. indicated that GD3, a core protein that initiates autophagy, is strongly associated with MAMs. They speculated that GD3 may be enriched in MAMs lipid rafts under autophagy stimulation, promoting autophagosome formation, and playing a role in early autophagy (58).

**Figure 1:**
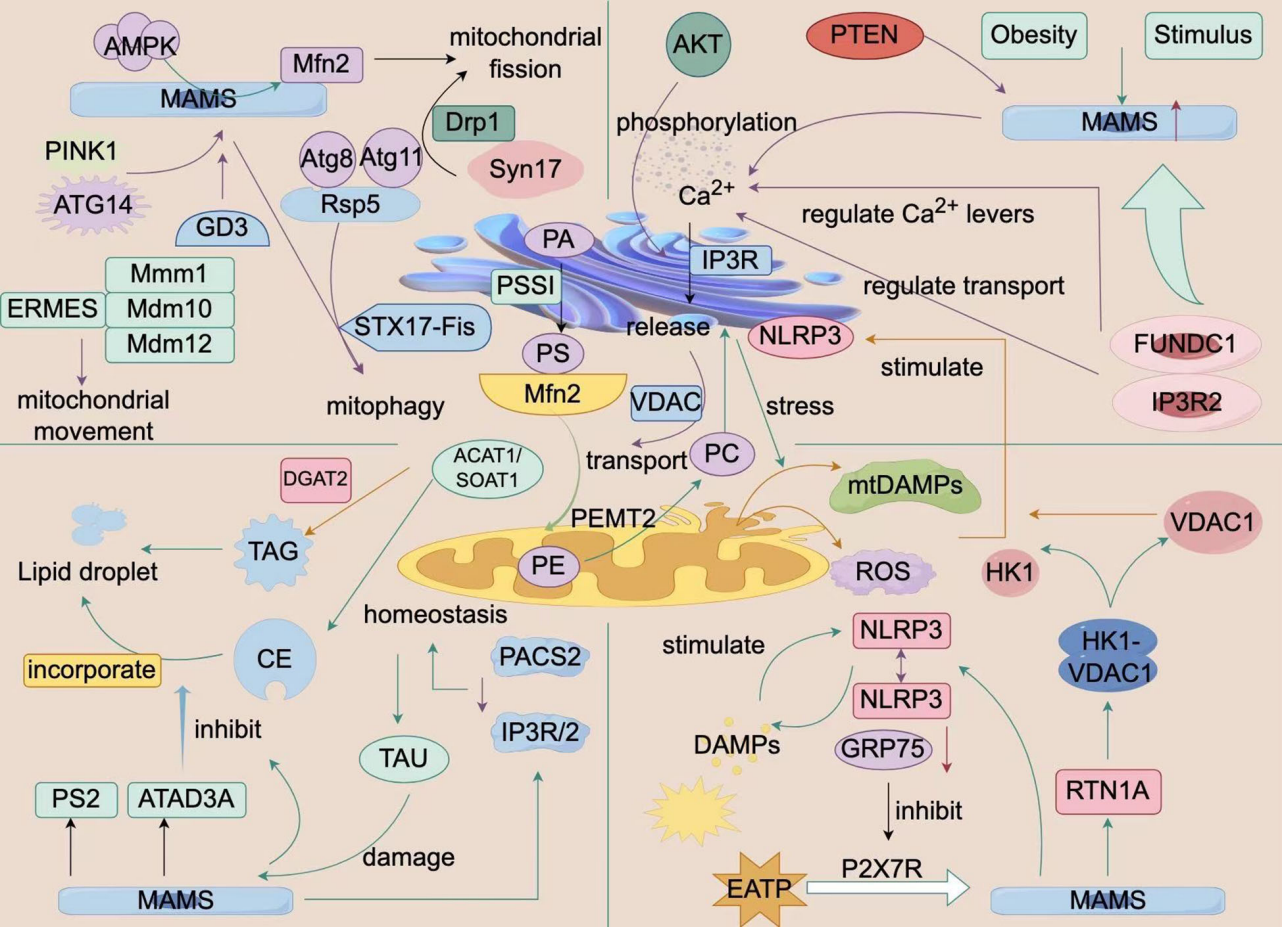
Biological Processes of ER-Mitochondria Crosstalk. This figure is a schematic diagram of the biological processes of interaction between the endoplasmic reticulum and mitochondria within a cell. It shows a variety of molecules and signaling pathways, including mitochondrial fission and fusion-related proteins (e.g., Mfn2, Drp1), autophagy-related proteins (e.g., PINK1, PARKIN), calcium ion signaling, and ER-related proteins (e.g., ERMES, VDAC1). These molecules and signaling pathways interact to regulate cellular metabolism, energy balance, and autophagy processes. Additionally, the figure illustrates the impact of external factors such as obesity and stimulation on these processes. The structures and molecules in the figure represent the biological processes of ER-mitochondria crosstalk (MAMs). The main structural molecules are described as follows: AMPK: AMP-activated protein kinase; MAMs: Mitochondria-associated membranes; PINK1: PTEN-induced putative kinase 1; ATG14: Autophagy-related protein 14; GD3: Disialyl ganglioside; ERMES: Endoplasmic reticulum-mitochondria contact site complex; STX17-Fis: Synaptophysin 17-mitochondrial fission protein 1 complex; PACS2: Phosphorylated protein sorting and transport protein 2; TAU: Microtubule-associated protein Tau; CE: Cholesterol ester; PS2: Presenilin 2; ATAD3A: ATPase family AAA domain-containing protein 3A; NLRP3: NOD-like receptor pyrin domain-containing protein 3; RTN1A: Reticulon 1A; FUNDC1: FUN14 domain-containing protein 1; VDAC1: Voltage-dependent anion channel 1; Mfn2: Mitofusin 2. The image is created with Figdraw.

During acute nutrient deficiency, ERMCS can alter its structure to dynamically enhance the transfer of calcium ions (Ca^2+^) and lipids from the ER to the mitochondria, stimulate apoptosis, and promote oxidative phosphorylation (30, 31). Mitochondrial Ca^2+^ overload is a key trigger of apoptosis, with MAMs protein complexes (e.g., IP3Rs, VDAC1) directly involved by promoting Ca^2+^ transmembrane transport. Studies also indicate that Bcl-2 family protein Bok amplifies Ca^2+^ signaling by binding to IP3Rs (33, 34), while PACS-2 deficiency reduces Ca^2+^ flux and inhibits apoptosis by disrupting ER-mitochondria connections (35). Notably, neuronal apoptosis is closely linked to ER-mitochondria crosstalk dysfunction, marked by disorders of proteins, such as Grp75 and Sigma1R, and excessive release of ROS and pro-inflammatory factors. Interventions in calcium homeostasis or the suppression of inflammatory responses can significantly restore neuronal function (59). In summary, ER-mitochondrial crosstalk, which integrates calcium homeostasis, lipid metabolism, and inflammatory responses, serves as a central regulator of cell autophagy and apoptosis (**Figure 1**; **Table 1**).

**Table 1:** Biological Processes of ER-Mitochondria Crosstalk.

Enzyme/ protein	Function	Author	References
PSS1	catalyze PS synthesis and participate in lipid synthesis	Vance JE et al.	(48)
PEMT2	the key enzyme of PC synthesis and involve in lipid synthesis	Vance JE et al.	(48, 118)
Mfn2	transport PS and activate protective mitophagy	Hernández-Alvarez et al.	(25, 78)
DGAT2	catalytic synthesis of triacylglycerol and associate with abnormal lipid metabolism	Sironi L et al.	(16)
ACAT1/SOAT1	catalyze the formation of cholesterol esters and relate to lipid droplet formation and abnormal lipid metabolism	Sironi L et al.	(16)
PS2	correct lipid droplet overdose in familial Alzheimer ‘s disease	Rossini M et al.	(119)
ATAD3A	inhibition of CYP46A1-mediated brain cholesterol metabolism	Zhao Y et al.	(120)
CYP46A1	mediate brain cholesterol metabolism	Zhao Y et al.	(120)
TAU	mediate endoplasmic reticulum-mitochondrial coupling and cholesterol transfer	Szabo L et al.	(121)
PACS2	regulate lipid homeostasis and affect anti-mitochondrial oxidation and affect insulin sensitivity	Arruda AP et al.	(21)
NLRP3	inflammasome,mediate immune response and inflammatory response	Christgen S et al.	(122, 123)
RTN1A	knockdown of HK1-VDAC1 interaction and trigger NLRP3 inflammasome activation	Xie Y et al.	(124)
GRP75	impact on the aggregation of NLRP3 inflammasome induced by the EATP-P2X7R pathway	Zhang JR et al.	(125)
IP3R、IP3R1/2	affect anti-mitochondrial oxidation ability and insulin sensitivity,mediate endoplasmic reticulum Ca^2+^ release,induce mitochondrial dysfunction and promote NLRP3 inflammasome activation	Arruda AP et al.	(21, 126)
PML	inhibit the NLRP3 inflammasome activation and affect the inflammatory response	Missiroli S et al.	(127)
OPTN	overexpression inhibits NLRP3 inflammasome expression and enhances mitophagy	Chen K et al.	(128)
FUNDC1	regulate Ca^2+^ content and affect mitochondrial fission	Wu S et al.	(24, 51)
VDAC	trigger NLRP3 inflammasome activation and effect the transfer of Ca^2+^ to the mitochondrial membrane space	Xie Y et al.	(13, 124)
PTEN	affect the release of Ca^2+^ in endoplasmic reticulum and regulate mitochondrial Ca^2+^ homeostasis and Ca^2+^-mediated apoptosis	Bononi A et al.	(129)
Akt	phosphorylated IP3R and inhibit its - mediated endoplasmic reticulum Ca^2+^ release	Marchi S et al.	(130)
Sig-1R-BiP Complex	regulate endoplasmic reticulum-mitochondrial Ca^2+^ signal transduction	Hayashi T et al.	(44)
SERCA2b	promote the influx of Ca^2+^ into endoplasmic reticulum and mitochondria	Gutiérrez T et al.	(45)
Bcl-2	reduce mitochondrial Ca^2+^ release and inhibit apoptosis	Foyouzi-YoussefiR et al.	(18)
PDZD8	mediates the transformation of Ca^2+^ from endoplasmic reticulum to mitochondria	Hirabayashi Y et al.	(22)
Drp1	mediates mitochondrial fission and autophagy	Kalia R et al.	(26, 47)
MFF	affect mitochondrial division and lack of mitochondrial contraction site changes	Kalia R et al.	(47, 131)
Syn17	regulate the activity and localization of Drp1 to promote mitochondrial division	Arasaki K et al.	(49)
AMPK	interact with Mfn2 to promote mitochondrial division	Hu Y et al.	(50)
MdmB	relate to mitochondrial division, down-regulation lead to mitochondrial division disorder	Garrido-Bazán V et al.	(132)
Mmm1	involve in mitochondrial polarization movement and mediate Ca^2+^ transport	Nguyen TT et al.	(22, 38)
Mdm10	participate in mitochondrial polarization movement and maintain mitochondrial function and homeostasis	Nguyen TT et al.	(38, 53, 133)
Mdm12	participate in mitochondrial polarization movement and maintain mitochondrial function and homeostasis	Nguyen TT et al.	(38, 52, 53)
Mdm34	participate in mitophagy	Mao K et al.	(56)
ATG14	participate in autophagosome formation	Hamasaki M et al.	(54)
PINK1	promote the formation of autophagosomes and MAMs	Yao RQ et al.	(55)
Beclin1	promote the formation of autophagosomes and MAMs	Yao RQ et al.	(55)
Atg8	participate in mitophagy	Mao K et al.	(56)
Atg11	participate in mitophagy	Mao K et al.	(56)
Fis1	inhibit STX17 metastasis and mitophagy	Jetto CT et al.	(27)
STX17	participate in inducing mitophagy	Jetto CT et al.	(27)
GD3	associate with MAMs and promote the formation of autophagic vacuoles	Böckler S et al.	(57)
Bok	bind IP3Rs promote Ca^2+^ transport from endoplasmic reticulum to mitochondria and affect apoptosis	Giorgi C et al.	(33, 34)
PACS-2	mediate endoplasmic reticulum-mitochondrial interference and promote apoptosis	Simmen T et al.	(35)

## 4 ER-mitochondria crosstalk in atherosclerosis

**Figure 2:**
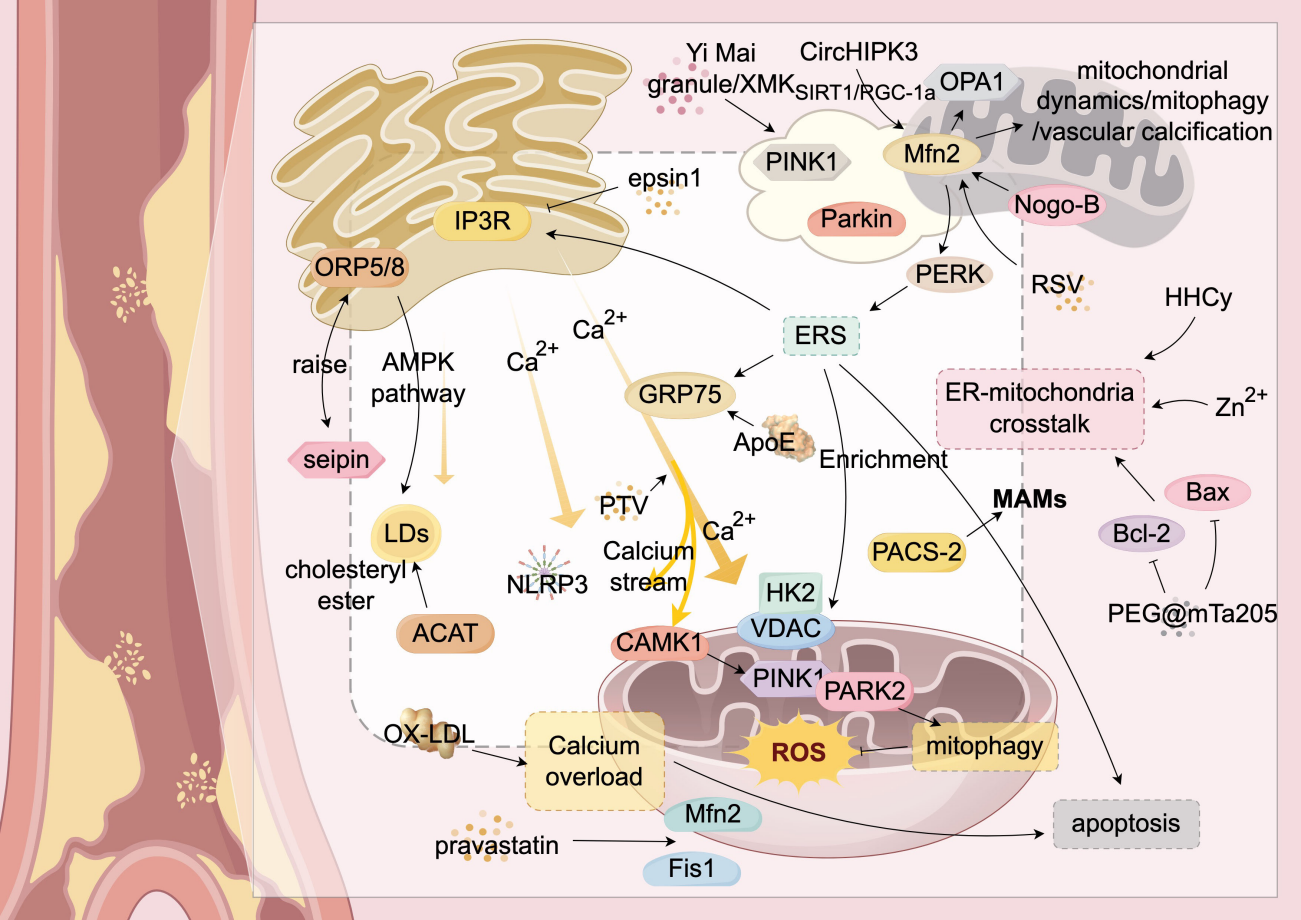
ER-Mitochondria Crosstalk in Atherosclerosis. This figure provides a detailed illustration of the complex mechanisms by which the endoplasmic reticulum (ER) and mitochondria interact through mitochondria-associated membranes (MAMs) and their associated proteins during atherosclerosis. It highlights key molecules and signaling pathways, including calcium ion flux and lipid transfer. Mfn2 serves as a central hub for communication between the ER and mitochondria. The figure also shows the roles of various proteins (such as ORP5/8, IP3R, GRP75, PERK, PAC5-2, VDAC, and Mfn2) in these interactions. Additionally, it illustrates abnormal biological processes such as ER stress (ERS), mitochondrial autophagy (mitophagy), and apoptosis, which collectively impact the progression of atherosclerosis. Through the interplay of these molecules and signaling pathways, ER-mitochondria crosstalk plays a critical role in the development of atherosclerosis. Structures and Molecules in the Figure: White rectangular areas: Mitochondria-associated membranes (MAMs); Yellow structure (upper left): Endoplasmic reticulum (ER); Yellow structures (upper right and lower right): Mitochondria; Yellow arrow (center): Calcium ion flow between the ER and mitochondria. The main structural molecules are described as follows: PINK1: PTEN-induced putative kinase 1; Parkin: Parkin protein; Mfn2: Mitofusin 2; Nogo-B: Reticulon 4B; Bax: Bcl-2-associated X protein; Bcl-2: B-cell lymphoma-2; Fis1: Mitochondrial fission protein 1; OX-LDL: Oxidized low-density lipoprotein; Seipin: Fat differentiation-related protein; ACAT: Acyl-CoA:cholesterol acyltransferase; ORP5/8: Oxysterol-binding protein-related proteins 5/8; GRP75: Glucose-regulated protein 75; CAMK1: Calcium/calmodulin-dependent protein kinase 1; NLRP3: NOD-like receptor pyrin domain-containing protein 3; LDs: Lipid droplets; PERK: RNA-dependent protein kinase-like ER kinase. The image is created with Figdraw.

In the pathological environment of AS, the ER and mitochondria form a dynamic signaling network through multiple mechanisms. This network involves various biological processes, including calcium homeostasis, lipid transport homeostasis, and enrichment and activation of key MAMs proteins, thereby accelerating the progression of AS. Elucidating these mechanisms is crucial for understanding AS pathogenesis and developing new diagnostic and therapeutic targets. Herein, we summarize and critically analyze the basic forms of ER-mitochondrial crosstalk in the context of AS (**Figure 2**; **Table 2**), highlighting the potential key role of the Mfn2 family of proteins in this process. However, the specific underlying mechanisms remain to be explored.

**Table 2:** Mechanisms of ER-Mitochondria Signaling Crosstalk in Atherosclerosis.

Mechanism	Function	Author	References
Inter-organelle Ca^2+^flux changes	Ca^2+^ overload impairs mitochondria, reducing ATP and increasing ROS, triggering apoptosis.	Marchi S et al.	(60)
	GRP75 mediates the physical interaction between VDAC1 and IP3R1.	Danese A et al.	(61)
	Ox-LDL induces PACS-2 enrichment, mediating cytochrome c release and endothelial apoptosis.	Yu S et al.	(62)
	LDL L5 subtype affects mitochondrial permeability, causing endothelial mitochondrial dysfunction.	Chen WY et al.	(63)
	PTV activates CAMK1 and recruits PARK2, enhancing mitophagy for ROS clearance and mitochondrial homeostasis.	Jie Yang et al.	(57)
	Nogo-B/Mfn2 interaction boosts ER-mitochondrial Ca^2+^ transport, promoting AS.	Zhang et al.	(64)
	Epsin1 degrades IP3R1, causing Ca^2+^ imbalance and promoting inflammation and AS.	Dong et al.	(66)
	HK2 dissociation activates IP3R, releases Ca^2+^, and oligomerizes VDAC, aiding NLRP3 inflammasome formation.	Baik SH et al.	(65)
Lipid transport and metabolism	The ER and mitochondria regulate lipid transport, influencing cholesterol, lipid droplet formation, and autophagy.	Santos NC et al.	(67)
	ORP5 complex interacts with seipin at MAMs-LD to regulate LD biogenesis.	Galmes R et al.	(70, 71)
	ORP8 regulates interactions with LC3 and GABARAPs via the AMPK pathway, affecting autophagy recognition and LD degradation.	Pu M et al.	(72)
	High cholesterol boosts MAMs in macrophages; pravastatin upregulates MAMs proteins, reducing ER-mitochondria contact.	Assis LHP et al.	(74)
	PEMT downregulation reduces plasma cholesterol and triglycerides, preventing diet-induced AS.	Li J et al.	(76)
Mfn2 protein acts as a potential link for signaling crosstalk	Mfn2 collaborates with OPA1 on the inner membrane to mediate mitochondrial fusion, maintaining mitochondrial dynamics.	Chandhok G et al.	(77)
	Mfn2 interacts with PERK, activating the PERK/eIF2α/ATF4 pathway and linking mitochondrial autophagy with ER stress.	Cao Y et al.	(6)
	PINK1 and Parkin affect ER-mitochondria contact and signaling.	Gelmetti V, Hu Y et al.	(56, 78)
	Nogo-B/Mfn2 interaction reduces ER-mitochondria contacts and Ca^2+^ flux, inhibiting mitochondrial ROS generation under inflammatory conditions.	Zhang Y et al.	(64)
	AIBP enhances PARK2 binding to Mfn1/Mfn2, promoting ubiquitination and mitophagy to inhibit ROS and apoptosis.	Choi SH et al.	(134)
Other	PEG@mTa2O5 downregulates Bcl-2 and Bax levels.	Jiao et al.	(79)
	Hcy enhances ER-mitochondria coupling, regulates ROS production, and promotes AS progression.	Feng J et al.	(80)
	Zn^2+^ treatment of cardiomyocytes upregulates PML, GRP78, etc., with unique effects on AS progression.	Dabravolski SA et al.	(81)
	HHCy upregulates DNMT1 and methylates Mfn2, promoting vascular smooth muscle cell proliferation.	Xu et al.	(82)
	MAMs-resident proteins amplify ROS signals via Ca^2+^ flux and redox reactions, exacerbating endothelial oxidative damage.	Xie H, Zhao J et al.	(83, 84)
	Under stress, ER stress sensors dissociate from BiP/GRP78, activating downstream apoptotic pathways.	Guo W et al.	(85)
	TMAO upregulates GRP78 to induce ER stress, promoting apoptosis in endothelial and smooth muscle cells.	Mohammadi A et al.	(86)
	PACS-2 knockout reduces ER-mitochondria contact sites, increasing VSMC apoptosis and accelerating late AS plaque destabilization.	Moulis et al.	(87)

### 4.1 Crosstalk between organelles based on calcium ion flux

Calcium ion flux between the endoplasmic reticulum (ER) and mitochondria is a key signaling pathway mediating organelle communication. The ER, acting as a cellular calcium reservoir, releases Ca^2+^ into the cytosol upon cellular demand, thereby dynamically regulating mitochondrial Ca^2+^ homeostasis. However, mitochondrial calcium overload directly inhibits basic mitochondrial function, leading to reduced ATP production and increased ROS generation, and can even trigger mitochondrial apoptosis (60). Within the cell, Ca^2+^ is released from the ER through IP3R and is transferred to the mitochondria via voltage-dependent anion channels (VDAC) on the outer mitochondrial membrane (OMM). VDAC1 physically connects to IP3R1 through GRP75, a MAM-associated protein, allowing GRP75 to directly promote calcium transfer from the ER to the mitochondria (61). During early ER stress, MAM proteins accumulate, increasing the ER-mitochondrial contact sites and accelerating mitochondrial calcium uptake to mitigate ER stress. This highlights the close collaborative relationship between the ER and mitochondria, with Ca^2+^ flux serving as the fundamental pathway for communication.

ER-mitochondrial interactions based on Ca^2+^ flux also play a crucial role in the progression of AS. Early AS lesions involve ox-LDL-induced endothelial cell apoptosis, a process linked to mitochondrial Ca^2+^ overload. PACS-2, enriched in MAM regions, promotes the formation of more ER-mitochondria contact sites under ox-LDL induction. This accelerates Ca^2+^ flux between the two organelles, leading to mitochondrial membrane potential loss and increased ROS production. These changes promote cytochrome c release and endothelial cell apoptosis (62). Additionally, the L5 subtype of LDL containing Apolipoprotein E (ApoE) interacts with VDAC, increasing mitochondrial permeability and causing mitochondrial dysfunction in endothelial cells (63). Yang et al. (57) demonstrated that pitavastatin (PTV) promotes calcium release from mitochondria to activate CAMK1, increasing the phosphorylation of PINK1. PINK1, in turn, recruits and phosphorylates PARK2 on the mitochondrial membrane, thereby activating mitophagy. This process is beneficial for ROS clearance and mitochondrial homeostasis in endothelial cells. Additionally, Zhang et al. (64) found that in ApoE−/− mice lacking Reticulon 4B(Nogo-B), Mfn2 protein levels decrease, and the development of AS lesions is inhibited. Further research indicated that Nogo-B interacts with Mfn2 in endothelial cells, increasing ER-mitochondria Ca^2+^ transport via ER-mitochondrial crosstalk. This process promotes ROS generation and activates the ROS-p38-p65 signaling pathway, enhancing inflammatory responses and promoting AS development. Collectively, these studies show that the Ca^2+^ flux between the ER and mitochondria in AS regulates mitochondrial homeostasis, ROS clearance, and apoptosis in endothelial cells, representing a specific mechanism by which ER-mitochondria crosstalk promotes AS progression.

Activation of the NLRP3 inflammasome in macrophages within plaques has also been linked to Ca^2+^ flux homeostasis in MAMs. A previous study revealed that NLRP3 inflammasome formation occurs as follows: hexokinase 2 (HK2) dissociates from VDAC on the outer mitochondrial membrane, activating IP3R signaling and causing Ca^2+^ release from the ER into the mitochondria. Increased mitochondrial calcium concentrations induce VDAC oligomerization, setting the stage for NLRP3 inflammasome formation (65). Furthermore, Dong et al. (66) found that epsin1 accelerates the ubiquitin-dependent degradation of IP3R1, leading to abnormal ER-mitochondrial calcium signaling and cytosolic free calcium imbalance, thereby promoting inflammation and AS. Thus, Ca^2+^ flux between the ER and MAMs also regulates inflammatory responses within AS plaques.

### 4.2 Lipid transport and metabolism as a crosstalk pathway

AS, a lipid metabolism-related disease, involves ER mitochondrial lipid transport via MAMs contact sites, including cholesterol esterification, lipid droplet formation, and autophagy (67). ORP5/8 proteins, key members of the oxysterol-binding protein-related protein (Osh/ORP) family, are the only proteins of the ORP family that are anchored to the ER membrane via their C-terminal transmembrane domains (68, 69). Studies have revealed that ORP5/8 proteins are critically involved in ER-mitochondria lipid trafficking (70). Specifically, the ORP5 complex localizes to MAMs subdomains and interacts with seipin to recruit it to MAMs-lipid droplet (LD) contact sites, thereby promoting LD biogenesis (71). In contrast, ORP8 regulates the interactions between LC3 and GABARAPs through the AMP-activated protein kinase (AMPK) pathway, thereby facilitating autophagic recognition and LD degradation (72). Although the homeostatic mechanisms of lipid transport homeostasis have been well studied, their molecular-level realization remains unclear. Scholars have determined the ORP8 domain crystal structure when bound to “cargo” lipids, using computer simulations to identify PS and PI4P binding to ORP8 (73), highlighting the unique importance of the “lid” structure for ORP8 function.

Hypercholesterolemia, a key risk factor of atherosclerosis, increases the number of MAMs contact sites in macrophages. Statins such as pravastatin upregulate MAM-related proteins such as Mfn2 and Fis1, also reducing ER-mitochondrial interactions and hinting at the link between ER-mitochondrial crosstalk and lipid metabolism/transport (74). Moreover, MAMs host key enzymes for cholesterol metabolism balance, such as acyl-CoA cholesterol acyltransferase (ACAT), which esterifies free cholesterol for storage in lipid droplets. ACAT dysfunction can also facilitate the development of AS (75). Downregulation of the MAMs protein PEMT also lowers plasma cholesterol and triglyceride levels, thereby preventing diet-induced AS (76). In summary, ER-mitochondrial lipid transport plays multiple roles in AS development, from cholesterol esterification and transport to triglyceride synthesis and lipid metabolism-protein regulation. The dysfunction of these processes may promote the formation of AS. Understanding these molecular mechanisms can help to clarify AS’s pathophysiology of AS and underpin new therapeutic approaches.

### 4.3 Mfn2 protein: a potential link for ER-mitochondria crosstalk

From the existing literature, we found that Mfn2 is a key link in the ER–mitochondrial crosstalk in AS pathology. The process of mitochondrial fission and fusion necessitates the recruitment of Drp1, Optic atrophy 1 (OPA1) and Mfn2 (77).Mfn2 on the mitochondrial outer membrane interacts with OPA1 on the inner membrane to promote mitochondrial fusion and maintain mitochondrial dynamics (77). Moreover, Mfn2 interacts with PERK protein, activating the PERK/eIF2α/ATF4 pathway upstream of UPR, thus linking mitochondrial autophagy and ER stress (6). Recent studies have expanded our understanding of the role of the PINK1/Parkin pathway, which mediates mitophagy. PINK1 and Parkin proteins, found in MAMs (56), interact with ER-mitochondrial communication proteins such as Mfn2 (78), promoting ER-mitochondrial contact and crosstalk. Furthermore, Nogo-B protein expression is upregulated in carotid/coronary atherosclerotic plaques. The Nogo-B protein stabilize Mfn2 to underpin ER-mitochondrial connections and Ca^2+^ flux homeostasis, thereby suppressing mitochondrial ROS generation during inflammatory responses (64). Thus, Mfn2, which intersects with multiple pathways, is a central hub protein in the ER-mitochondrial interaction network in AS.

### 4.4 Other factors

Advances in nanotechnology have enabled the use of various nanoparticles in disease diagnosis, bioimaging, and drug delivery. However, long-term exposure to nanoparticles can damage endothelial cells and exacerbate AS. Jiao et al. (79) found that the mesoporous tantalum oxide nanomaterial PEG@mTa_2_O_5_ downregulates Bcl-2 and Bax expression. While Bcl-2 family proteins are linked to ER-mitochondria crosstalk in apoptosis, whether PEG@mTa_2_O_5_ worsens AS via this crosstalk remains unclear.

Hyperhomocysteinemia (HHCy) contributes to AS progression. Homocysteine (Hcy) increases ER-mitochondrial coupling and promotes mitochondrial ROS production, which can disrupt Ca^2+^ homeostasis and alter membrane potential. This reprograms mitochondrial metabolism and activates T cells, driving AS progression through cytokine/chemokine release, immune responses, and Treg regulation (80). Zn^2+^ homeostasis also promotes AS progression via ER-mitochondria crosstalk. Treating cardiomyocytes with Zn^2+^ upregulates ER-mitochondria contact proteins like PML, ER stress proteins such as GRP78, and calmodulin (81). Epigenetic regulation studies further suggest that HHCy promotes AS via ER-mitochondrial crosstalk. Xu et al. (82) found that HHCy upregulates DNMT1 and increases Mfn2 methylation. Downregulation of Mfn2 in AS plaques drives abnormal vascular smooth muscle cell proliferation (82).

Excessive ROS generation is associated with several cardiovascular diseases, including AS. MAMs serve as key hubs for ROS production. Mitochondrial cytochrome b5, a target of cytochrome c, activates the CYP-dependent monooxygenase system, thereby increasing ROS production. MAM-resident proteins (e.g., GRP75, ERO1, SIG-1R, and VDAC) amplify ROS signals by promoting Ca^2+^ flux and redox reactions, and exacerbating endothelial cell oxidative damage (83, 84).

ER stress integrates with mitochondrial apoptotic signals via MAMs and is directly involved in AS pathology. Under stress, ER stress sensors (PERK and IRE1) dissociate from the chaperone BiP/GRP78, activating downstream apoptotic pathways (85). Trimethylamine N-oxide (TMAO), an AS risk factor, induces ER stress by upregulating GRP78 and promoting the apoptosis of endothelial and smooth muscle cells (86). MAM dysfunction has also been linked to the loss of AS plaque stability. Moulis et al. found that PACS-2 knockout reduces ER-mitochondrial contact sites, which increased vascular smooth muscle cell (VSMC) apoptosis, and accelerated late-stage AS plaque instability (87).

Research indicates that age-related cardiovascular diseases, including AS, are closely related to mitochondrial dysfunction and abnormal ER-mitochondrial interactions. Specifically, the MAM protein PACS-2 inhibits apoptosis in vascular endothelial and smooth muscle cells, promoting age-related AS pathology (87, 88). Additionally, mtDNA mutations in Mfn2 can impair MAMs functions such as phospholipid synthesis/transport and calcium homeostasis, thereby reducing ER-mitochondria contact sites (89). Similarly, Granatiero et al. (90) observed that the 13514A>G mtDNA mutation decreased ER-mitochondrial contact sites in MELAS cells (primary skin fibroblasts derived from MELAS or Leigh syndrome patients), accompanied by blocked calcium ion flow and reduced mitochondrial calcium uptake. However, these mtDNA mutations have not been confirmed in AS. Given that mitochondrial dysfunction and mitochondrial DNA mutations are characteristics of mitochondrial aging, combined with the aforementioned research findings, does mitochondrial aging promote age-related atherosclerotic (AS) progression by disrupting ER-mitochondria crosstalk? This represents a promising avenue for future research.

## 5 Targeting ER-mitochondria crosstalk in AS: promising drugs

**Figure 3:**
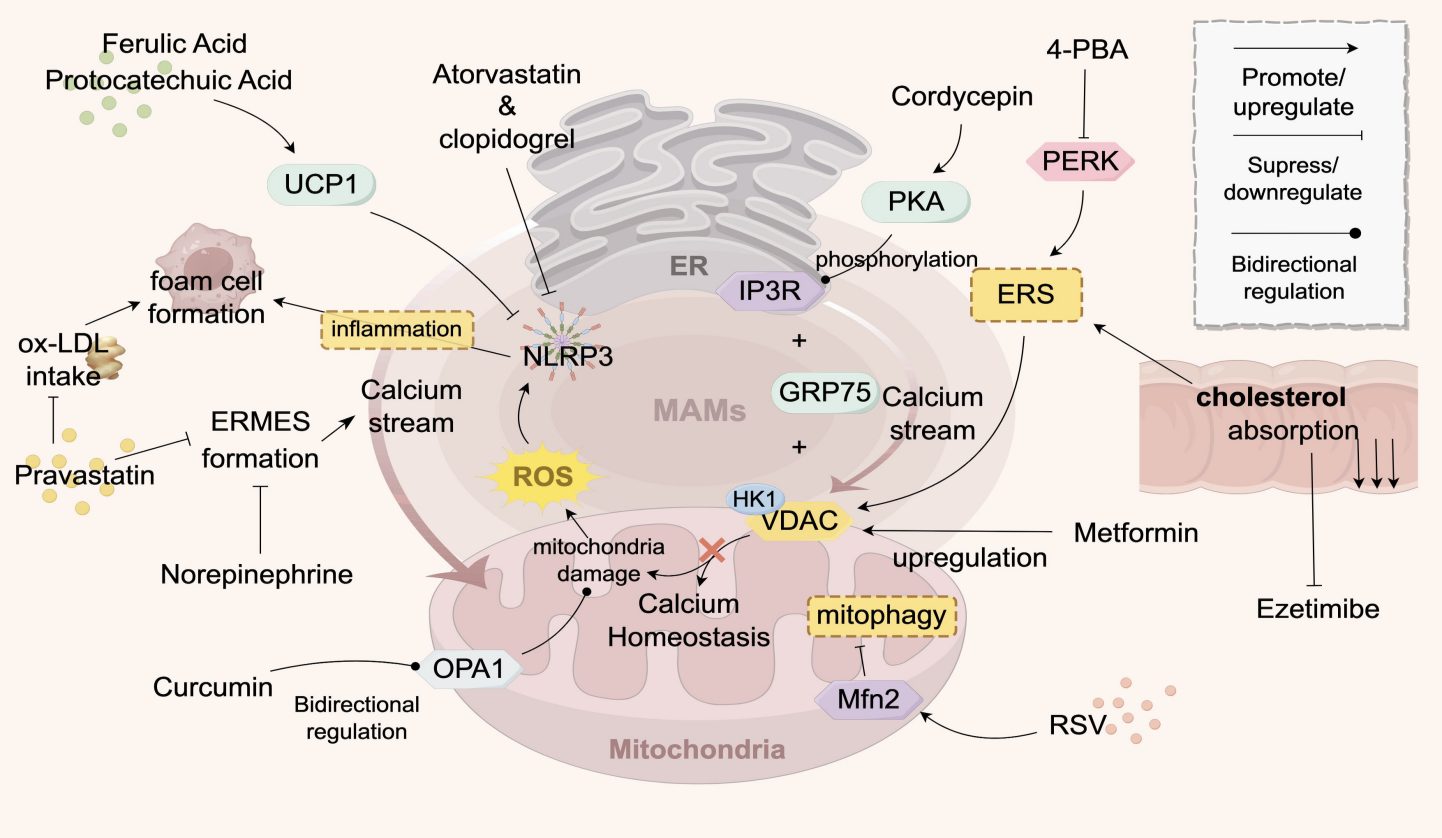
Drugs Targeting ER-Mitochondria Crosstalk in AS. This figure illustrates the signaling exchange between the ER and mitochondria via MAMs contact sites in an atherosclerotic environment. The ER transfers Ca^2+^ to mitochondria to maintain their function, but Ca^2+^ overload can inhibit mitochondrial function, reducing ATP and increasing ROS, which triggers apoptosis. Statins stabilize endothelial function by inhibiting a key enzyme in cholesterol synthesis. Various drugs (e.g., pravastatin, atorvastatin, 4-PBA) target ER-mitochondria crosstalk to improve calcium ion flow, maintain mitochondrial function, reduce inflammation, and decrease foam cell formation, thereby improving AS. Traditional Chinese medicine components, such as curcumin and resveratrol, modulate key proteins (e.g., OPA1, MFN2) to influence mitochondrial function and show therapeutic potential. The structures and molecules in the figure represent the targets of AS drugs that act on ER-mitochondria crosstalk. The gray structure at the top represents the ER, the pink structure at the bottom represents the mitochondrion, and the gray-pink area between them represents the MAMs. The meanings of the various arrows are labeled in the legend in the top right corner of the figure. The main structural molecules are described as follows: UCP1: Uncoupling protein 1; ROS: Reactive oxygen species; OPA1: Optic atrophy protein 1; NLRP3: NOD-like receptor pyrin domain-containing protein 3; VDAC: Voltage-dependent anion channel; HK1: Hexokinase 1; PERK: Protein kinase R-like ER kinase; PKA: Protein kinase A; RSV: Resveratrol; ERS: Endoplasmic reticulum stress; GRP75: Glucose-regulated protein 75; IP3R: Inositol 1,4,5-trisphosphate receptor; 4-PBA: 4-phenylbutyric acid. The image is created with Figdraw.

A number of pharmaceutical reagents target ER-mitochondrial crosstalk through distinct mechanisms (**Figure 3**; **Table 3** ), demonstrating their potential for treating cardiovascular diseases, including AS.

**Table 3:** Drugs Targeting ER-Mitochondria Crosstalk in AS.

Name	Therapeutic Mechanism	Category	References
Pravastatin	Pravastatin reverses changes in ER-mitochondria contact points, restoring ER-mitochondria interactions. It also reduces the uptake of oxidized low-density lipoprotein (ox-LDL), decreases foam cell formation, and alleviates AS progression by reducing oxidative stress and inflammatory responses.	Statins	(74)
4-PBA	4-PBA enhances plaque stability, inhibits oxidative stress and apoptosis, reduces the expression of ER stress-related proteins, and enhances the expression of the CLOCK protein in mice.	ER Stress Inhibitor	(91, 92, 135)
Atorvastatin	When used in combination with magnesium hydroxide, it enhances lipid-lowering effects and is used as a high-intensity statin to reduce LDL-C.	Statins	(102)
Ezetimibe	Inhibits Niemann-Pick C1-like 1 protein, blocking the mammalian target of rapamycin pathway and inducing apoptosis.	Cholesterol Absorption Inhibitor	(97)
Metformin	Binds to the VDAC1 target, improving conditions such as diabetes, COVID-19, cancer, neurodegenerative diseases, and aging.	Antidiabetic Agent	(94)
Ferulic Acid, Protocatechuic Acid	Upregulates UCP1 in adipose tissue, inhibiting the NLRP3-IL-1β inflammatory pathway and foam cell formation in AS plaques.	Nonsteroidal Anti-inflammatory Drugs	(101)
Clopidogrel	Reduces the activation of the NLRP3-IL-1β inflammatory pathway, thereby delaying the progression of atherosclerotic lesions.	Antiplatelet Drug	(104, 105)
Curcumin	Regulates OPA1 protein expression to modulate mitochondrial function, affecting the proliferation and migration of vascular endothelial cells. It also acts on the NF-κB signaling pathway, inhibiting the transmission of inflammatory signals to mitochondria, reducing oxidative stress, and stabilizing intracellular homeostasis to improve cardiovascular diseases and atherosclerosis.	Polyphenolic Compound	(106–108)
Cordycepin-WIB801C	Phosphorylates IP3R proteins on MAMs in a cAMP/A-kinase-dependent manner, inhibiting the mobilization of intracellular calcium levels ([Ca²⁺]i) to treat and prevent atherosclerosis.	Antiplatelet, Neuroprotective, Anti-inflammatory, Immunomodulator	(109)
Resveratrol	Alleviates oxidative stress and inflammation, enhances metabolic capacity, increases NO production, inhibits VSMC proliferation, and promotes autophagy to reduce atherosclerosis.	Polyphenolic Compound	(6, 110)
N-Acetylcysteine	Clears reactive oxygen species (ROS), alleviates ER stress and mitochondrial damage, and improves the function of vascular smooth muscle cells (VSMCs).	Antioxidant	(136)
Mitochondrial Calcium Uniporter Inhibitor	Inhibits mitochondrial calcium uptake, reducing mitochondrial dysfunction and cell proliferation.	Calcium Ion Regulator	(136)
Humanin	A mitochondrial-derived peptide that protects cells from oxidative stress and ER stress-induced cell death.	Mitochondrial Protectant	(137)
mdivi-1	Inhibits the mitochondrial fission protein DRP1, regulating mitochondrial dynamics and preventing phenotypic transitions in VSMCs to alleviate atherosclerosis.	Small Molecule Regulator	(139)
SIRT1 Activator	SIRT1, a deacetylase, modulates mitochondrial function and antioxidant responses, alleviating ER and mitochondrial stress.	Metabolic Regulator	(138)
Tauroursodeoxycholic Acid	Alleviates ER stress, providing potential protection against atherosclerosis.	Chemical Chaperone	(135)

Some drugs improve AS by inhibiting the Ca^2+^ flow between the ER and mitochondria. Pravastatin reduces ER-mitochondria interaction sites, inhibiting Ca^2+^ transfer and decreasing foam cell formation by reducing macrophage uptake of ox-LDL (81). Noradrenaline downregulates mitochondrial Ca^2+^ uptake and ER-mitochondrial coupling sites in cardiomyocytes (74). The chemical 4-Phenylbutyric acid (4-PBA) reduces ER stress levels, suppresses PERK activation to maintain Ca^2+^ homeostasis (91), and prevents adverse effects caused by excessive Ca^2+^ influx, such as mitochondrial membrane potential depolarization and increased ROS production (91–93). Metformin upregulates the expression of VDAC1 protein in MAMs (94), thereby preserving mitochondrial Ca^2+^ homeostasis and reducing ROS generation (95).

Several agents target lipid synthesis to ameliorate AS. Statins exert their therapeutic effects by inhibiting key enzymes involved in cholesterol synthesis, such as HMG-CoA reductase, thereby reducing cholesterol biosynthesis and indirectly suppressing lipid transport and signaling crosstalk between the ER and mitochondria (74). This mechanism stabilizes vascular endothelial cell function, attenuates foam cell formation, and ultimately slows AS progression (96). Ezetimibe reduces systemic cholesterol levels by inhibiting intestinal cholesterol absorption, thereby mitigating excessive cholesterol-induced ER stress and its detrimental effects on mitochondrial function (97, 98). Yimai granules improve AS by activating the Pink1-Mfn2-Parkin pathway via miRNA-125a-5p, which enhances mitophagy, suppresses proinflammatory factor release, inhibits vasoconstrictor production (99).

Moreover, emerging data indicate that certain agents can directly target MAMs to suppress inflammatory responses and are a platform for NLRP3 inflammasome activation, since reducing ER stress or MAM formation can inhibit NLRP3 (100). Ferulic acid and protocatechuic acid upregulate UCP1 in adipose tissue to inhibit the NLRP3-IL-1β pathway and foam cell formation (101). Atorvastatin inhibits the activation of the NLRP3 inflammasome, reduces the release of inflammatory factors, mitigates vascular inflammatory responses, and thereby suppresses the progression of atherosclerosis (102, 103). Clopidogrel delays the progression of atherosclerotic lesions by inhibiting the activation of the NLRP3-IL-1β inflammatory pathway (104, 105).

Specific herbal formulas and active components of traditional Chinese medicine (TCM) can also ameliorate AS by targeting the ER-mitochondrial crosstalk. Curcumin improves AS by modulating the expression OPA1, bidirectionally regulating mitochondrial function, thereby influencing the proliferation and migration of vascular endothelial cells (106). Additionally, curcumin acts on the NF-κB signaling pathway (107) by inhibiting the propagation of inflammatory signals to the mitochondria, reducing oxidative stress damage, stabilizing intracellular homeostasis, and lowering the risk of cardiovascular diseases and AS (108). Cordycepin (CE)-WIB801C suppresses AS progression via cAMP-dependent protein kinase (PKA)-mediated phosphorylation of the inositol trisphosphate receptor (IP3R) on MAMs, thereby inhibiting intracellular calcium mobilization ([Ca^2+^]i) (109). Resveratrol (RSV) upregulates the expression of *Mfn1* and *Mfn2* in MAMs (110), stabilizing mitochondrial dynamics and improving mitochondrial function. Mfn2, a key protein promoting ER-mitochondrial communication, modulates inter-organelle signaling crosstalk through its expression levels (6). Taken together, it appears that these TCM components target key MAM proteins such as OPA1, IP3R, Mfn1, and Mfn2.

## 6 Discussion

The ER and mitochondria exert profound effects on various physiological processes within cells through complex molecular mechanisms. However, crosstalk between the two organelles plays an even more critical role in maintaining lipid and calcium homeostasis, promoting inflammatory responses, and apoptosis. Exploring the ER-mitochondria crosstalk in the context of AS not only deepens our understanding of the pathophysiology of the disease, but also shifts the research focus from isolated organelles to their dynamic interactions, providing new insights for future studies.

Although research on ERMES-related proteins as biomarkers for AS remains limited, mtDNA abnormalities have been established as effective molecular markers for AS (111). Studies indicate that dysfunctional ERMES can induce excessive mitochondrial fission (e.g., Drp1-dependent fission), compromising mitochondrial membrane integrity and significantly increasing the risk of mtDNA leakage into the cytoplasm (112). Moreover, mitochondrial dynamic disorders reduce oxidative phosphorylation efficiency, leading to decreased ATP synthesis and reactive oxygen species (ROS) accumulation, further increasing the risk of mtDNA leakage (113). In clinical translation, the peripheral blood cell mitochondrial DNA copy number, owing to its high sensitivity and accessibility, has emerged as a promising indicator for early screening and risk assessment of AS (114). Whole-genome sequencing technology can precisely quantify fluctuations in mtDNA-CN, and its abnormal reduction has been linked to plaque instability and increased risk of cardiovascular events (111). Thus, molecules downstream of MAMs (e.g., mtDNA) show promise for transitioning from basic research to clinical applications. Future studies should investigate the potential of MAM-related proteins as biomarkers and explore the dynamic regulatory strategies targeting MAMs to disrupt the AS cycle.

The current clinical management of AS primarily relies on pharmacological and interventional therapies. Statins are commonly used lipid-lowering drugs that reduce blood cholesterol levels, stabilize plaques, and reduce the risk of cardiovascular events. Other medications, such as metformin and ezetimibe, also act through distinct mechanisms. Interventional therapies such as coronary artery stenting (115) and carotid endarterectomy (116), directly address vascular stenosis or occlusion and restore blood flow. However, these approaches carry risks including drug resistance, intolerance, side effects, and post-procedural complications such as restenosis and thrombosis (117).

Given these challenges, there is an urgent need to address the limitations of the existing therapies and explore novel approaches. The precise regulation of the ER-mitochondrial crosstalk in AS offers a new perspective. Key questions include ensuring therapeutic efficacy, enhancing safety, and identifying more effective and safer treatment strategies. To achieve this, further elucidation of the molecular mechanisms underlying the ER-mitochondrial crosstalk in AS, as well as the functions and interactions of specific MAMs proteins, is required. Identifying potential drug targets within MAMs and understanding the precise regulation of calcium signaling and lipid transport-related molecules will provide a robust foundation for drug design. Additionally, exploring the unique advantages and targets of TCM and its active components in modulating ER-mitochondrial crosstalk, combined with modern biotechnology, may unlock novel therapeutic potential. This may lead to the development of multi-target and highly efficacious anti-AS drugs. Moreover, the development of nanotechnology-based drug delivery systems could further enhance the targeted drug delivery to AS lesions. In summary, in-depth research on ER-mitochondria crosstalk in AS holds promise for revolutionizing cardiovascular disease prevention and treatment, addressing current diagnostic and therapeutic gaps, and offering safer and more effective strategies for patients with AS.

## References

[1] Perrotta I. Atherosclerosis: from molecular biology to therapeutic perspective 2.0. Int J Mol Sci. (2022) 23:15158. doi: 10.3390/ijms232315158 36499481 PMC9740737

[2] Copeland DE Dalton AJ. An association between mitochondria and the endoplasmic reticulum in cells of the pseudobranch gland of a teleost. J Biophys Biochem Cytol. (1959) 5:393–6. doi: 10.1083/jcb.5.3.393 PMC2224680 13664679

[3] Luan Y Luan Y Yuan RX Feng Q Chen X Yang Y. Structure and function of mitochondria-associated endoplasmic reticulum membranes (MAMs) and their role in cardiovascular diseases. Oxid Med Cell Longev. (2021) 2021:4578809. doi: 10.1155/2021/4578809 34336092 PMC8289621

[4] Janikiewicz J Szymański J Malinska D Patalas-Krawczyk P Michalska B Duszyński J. Mitochondria-associated membranes in aging and senescence: structure, function, and dynamics. Cell Death Dis. (2018) 9:332. doi: 10.1038/s41419-017-0105-5 29491385 PMC5832430

[5] Wang N Wang C Zhao H He Y Lan B Sun L. The MAMs structure and its role in cell death. Cells. (2021) 10(3):657. doi: 10.3390/cells10030657 33809551 PMC7999768

[6] Cao Y Chen Z Hu J Feng J Zhu Z Fan Y. Mfn2 regulates high glucose-induced MAMs dysfunction and apoptosis in podocytes via PERK pathway. Front Cell Dev Biol. (2021) 9:769213. doi: 10.3389/fcell.2021.769213 34988075 PMC8721005

[7] Song Z Song H Liu D Yan B Wang D Zhang Y. Overexpression of MFN2 alleviates sorafenib-induced cardiomyocyte necroptosis via the MAM-CaMKIIdelta pathway *in vitro* and *in vivo* . Theranostics. (2022) 12:1267–85. doi: 10.7150/thno.65716 PMC8771548 35154486

[8] Stone SJ Vance JE. Phosphatidylserine synthase-1 and -2 are localized to mitochondria-associated membranes. J Biol Chem. (2000) 275:34534–40. doi: 10.1074/jbc.m002865200 10938271

[9] Kornmann B Currie E Collins SR Schuldiner M Nunnari J Weissman JS. An ER-mitochondria tethering complex revealed by a synthetic biology screen. Science. (2009) 325(5939):477–81. doi: 10.1126/science.1175088 PMC2933203 19556461

[10] Okamoto M Nakano K Takahashi-Nakaguchi A Sasamoto K Yamaguchi M Teixeira MC. In ERMES component controls mitochondrial morphology, mtROS, and drug efflux pump expression, resulting in azole susceptibility. J Fungi. (2023) 9:240. doi: 10.3390/jof9020240 PMC9965728 36836353

[11] Wozny MR Di Luca A Morado DR Picco A Khaddaj R Campomanes P. In situ architecture of the ER-mitochondria encounter structure. Nature. (2023) 618:188–92. doi: 10.1038/s41586-023-06050-3 PMC7614606 37165187

[12] Jeong H Park J Jun Y Lee C. Crystal structures of Mmm1 and Mdm12-Mmm1 reveal mechanistic insight into phospholipid trafficking at ER-mitochondria contact sites. Proc Natl Acad Sci United States America. (2017) 114:E9502–11. doi: 10.1073/pnas.1715592114 PMC5692604 29078410

[13] Kornmann B Walter P. ERMES-mediated ER-mitochondria contacts: molecular hubs for the regulation of mitochondrial biology. J Cell Sci. (2010) 123:1389–93. doi: 10.1242/jcs.058636 PMC2858017 20410371

[14] Nguyen TT Lewandowska A Choi JY Markgraf DF Junker M Bilgin M. Gem1 and ERMES do not directly affect phosphatidylserine transport from ER to mitochondria or mitochondrial inheritance. Traffic. (2012) 13:880–90. doi: 10.1111/j.1600-0854.2012.01352.x PMC3648210 22409400

[15] Rasul F Zheng F Dong F He J Liu L Liu W. Emr1 regulates the number of foci of the endoplasmic reticulum-mitochondria encounter structure complex. Nat Commun. (2021) 12:521. doi: 10.1038/s41467-020-20866-x 33483504 PMC7822926

[16] Cheema JY He J Wei W Fu C. The endoplasmic reticulum-mitochondria encounter structure and its regulatory proteins. Contact (Thousand Oaks). (2021) 4:25152564211064491. doi: 10.1177/25152564211064491 37366373 PMC10243566

[17] Vance JE. Inter-organelle membrane contact sites: implications for lipid metabolism. Biol Direct. (2020) 15:24. doi: 10.1186/s13062-020-00279-y 33176847 PMC7661199

[18] Cui Z Vance JE Chen MH Voelker DR Vance DE. Cloning and expression of a novel phosphatidylethanolamine N-methyltransferase. A sp*ecific biochemical and cytological marker for a unique membrane fraction in rat liver* . J Biol Chem. (1993) 268:16655–63. doi: 10.1016/s0021-9258(19)85468-6 8344945

[19] Hernandez-Alvarez MI Sebastián D Vives S Ivanova S Bartoccioni P Kakimoto P. Deficient endoplasmic reticulum-mitochondrial phosphatidylserine transfer causes liver disease. Cell. (2019) 177:881–895.e17. doi: 10.1016/j.cell.2019.04.010 31051106

[20] Ganji R Paulo JA Xi Y Kline I Zhu J Clemen CS. The p97-UBXD8 complex regulates ER-Mitochondria contact sites by altering membrane lipid saturation and composition. Nat Commun. (2023) 14:638. doi: 10.1038/s41467-023-36298-2 36746962 PMC9902492

[21] Sironi L Restelli LM Tolnay M Neutzner A Frank S. Dysregulated interorganellar crosstalk of mitochondria in the pathogenesis of parkinson’s disease. Cells. (2020) 9:233. doi: 10.3390/cells9010233 31963435 PMC7016713

[22] Rossini M García-Casas P Filadi R Pizzo P. Loosening ER-mitochondria coupling by the expression of the presenilin 2 loop domain. Cells. (2021) 10:1968. doi: 10.3390/cells10081968 34440738 PMC8394530

[23] Zhao Y Hu D Wang R Sun X Ropelewski P Hubler Z. ATAD3A oligomerization promotes neuropathology and cognitive deficits in Alzheimer’s disease models. Nat Commun. (2022) 13:1121. doi: 10.1038/s41467-022-28769-9 35236834 PMC8891325

[24] Szabo L Cummins N Paganetti P Odermatt A Papassotiropoulos A Karch C. ER-mitochondria contacts and cholesterol metabolism are disrupted by disease-associated tau protein. EMBO Rep. (2023) 24:e57499. doi: 10.15252/embr.202357499 37401859 PMC10398652

[25] Arruda AP Pers BM Parlakgül G Güney E Inouye K Hotamisligil GS. Chronic enrichment of hepatic endoplasmic reticulum-mitochondria contact leads to mitochondrial dysfunction in obesity. Nat Med. (2014) 20:1427–35. doi: 10.1038/nm.3735 PMC4412031 25419710

[26] Christgen S Kanneganti TD. Inflammasomes and the fine line between defense and disease. Curr Opin Immunol. (2020) 62:39–44. doi: 10.1016/j.coi.2019.11.007 31837596 PMC7067632

[27] Missiroli S Patergnani S Caroccia N Pedriali G Perrone M Previati M. Mitochondria-associated membranes (MAMs) and inflammation. Cell Death Dis. (2018) 9:329. doi: 10.1038/s41419-017-0027-2 29491386 PMC5832426

[28] Bronner DN Abuaita BH Chen X Fitzgerald KA Nuñez G He Y. Endoplasmic reticulum stress activates the inflammasome via NLRP3- and caspase-2-driven mitochondrial damage. Immunity. (2015) 43:451–62. doi: 10.1016/j.immuni.2015.08.008 PMC4582788 26341399

[29] Xie YF Cai H Zhong F Xiao W Gordon RE. Reticulon-1A mediates diabetic kidney disease progression through endoplasmic reticulum-mitochondrial contacts in tubular epithelial cells. Kidney Int. (2022) 102:293–306. doi: 10.1016/j.kint.2022.02.038 35469894 PMC9329239

[30] Zhang JR Shen SY Zhai MY Shen ZQ Li W Liang LF. Augmented microglial endoplasmic reticulum-mitochondria contacts mediate depression-like behavior in mice induced by chronic social defeat stress. Nat Commun. (2024) 15:5199. doi: 10.1038/s41467-024-49597-z 38890305 PMC11189428

[31] Gao P Yang WX Sun L. Mitochondria-associated endoplasmic reticulum membranes (MAMs) and their prospective roles in kidney disease. Oxid Med Cell Longevity. (2020) 2020:3120539. doi: 10.1155/2020/3120539 PMC7487091 32952849

[32] Ye L Zeng Q Ling M Ma R Chen H Lin F. Inhibition of IP3R/ca2+Dysregulation protects mice from ventilator-induced lung injury endoplasmic reticulum and mitochondrial pathways. Front Immunol. (2021) 12:729094. doi: 10.3389/fimmu.2021.729094 34603302 PMC8479188

[33] Missiroli S Perrone M Gafà R Nicoli F Bonora M Morciano G. PML at mitochondria-associated membranes governs a trimeric complex with NLRP3 and P2X7R that modulates the tumor immune microenvironment. Cell Death Differentiation. (2023) 30:429–41. doi: 10.1038/s41418-022-01095-9 PMC9713080 36450825

[34] Chen KH Feng L Hu W Chen J Wang X Wang L. Optineurin inhibits NLRP3 inflammasome activation by enhancing mitophagy of renal tubular cells in diabetic nephropathy. FASEB J. (2019) 33:4571–85. doi: 10.1096/fj.201801749rrr 30571313

[35] Hirabayahi Y Kwon SK Paek H Pernice WM Paul MA Lee J. ER-mitochondria tethering by PDZD8 regulates Ca dynamics in mammalian neurons. Science. (2017) 358:623–9. doi: 10.1126/science.aan6009 PMC5818999 29097544

[36] López-Otín C Pietrocola F Roiz-Valle D Galluzzi L Kroemer G. Meta-hallmarks of aging and cancer. Cell Metab. (2023) 35:12–35. doi: 10.1016/j.cmet.2022.11.001 36599298

[37] Wu SN Lu Q Wang Q Ding Y Ma Z Mao X. Binding of FUN14 domain containing 1 with inositol 1,4,5-trisphosphate receptor in mitochondria-associated endoplasmic reticulum membranes maintains mitochondrial dynamics and function in hearts *in vivo* . Circulation. (2017) 136:2248–66. doi: 10.1161/circulationaha.117.030235 PMC5716911 28942427

[38] Foyouzi-Youssefi R. Bcl-2 decreases the free Ca2+ concentration within the endoplasmic reticulum. Proc Natl Acad Sci U S A. (2000) 97:5723–8. doi: 10.1073/pnas.97.11.5723 PMC18500 10823933

[39] Garrido-Bazán V Aguirre J. H_2_O_2_ induces calcium and ERMES complex-dependent mitochondrial constriction and division as well as mitochondrial outer membrane remodeling in. J Fungi. (2022) 8:829. doi: 10.3390/jof8080829 PMC9410301 36012817

[40] Sogo LF Yaffe MP. Regulation of mitochondrial morphology and inheritance by Mdm10p, a protein of the mitochondrial outer membrane. J Cell Biol. (1994) 126:1361–73. doi: 10.1083/jcb.126.6.1361 PMC2290945 8089171

[41] Yuan M Gong M He J Xie B Zhang Z Meng L. IP3R1/GRP75/VDAC1 complex mediates endoplasmic reticulum stress-mitochondrial oxidative stress in diabetic atrial remodeling. Redox Biol. (2022) 52:102289. doi: 10.1016/j.redox.2022.102289 35344886 PMC8961221

[42] Marchi S Marinello M Bononi A Bonora M Giorgi C Rimessi A. Selective modulation of subtype III IP(3)R by Akt regulates ER Ca(2)(+) release and apoptosis. Cell Death Dis. (2012) 3:e304. doi: 10.1038/cddis.2012.45 22552281 PMC3366079

[43] Bononi A Bonora M Marchi S Missiroli S Poletti F Giorgi C. Identification of PTEN at the ER and MAMs and its regulation of Ca(2+) signaling and apoptosis in a protein phosphatase-dependent manner. Cell Death Differ. (2013) 20:1631–43. doi: 10.1038/cdd.2013.77 PMC3824603 23811847

[44] Belgareh-Touzé N Cavellini L Cohen MM. Ubiquitination of ERMES components by the E3 ligase Rsp5 is involved in mitophagy. Autophagy. (2017) 13:114–32. doi: 10.1080/15548627.2016.1252889 PMC5240830 27846375

[45] Zhou H Wang S Hu S Chen Y Ren J. ER-mitochondria microdomains in cardiac ischemia-reperfusion injury: A fresh perspective. Front Physiol. (2018) 9:755. doi: 10.3389/fphys.2018.00755 29962971 PMC6013587

[46] Ikeda Y Shirakabe A Maejima Y Zhai P Sciarretta S Toli J. Endogenous Drp1 mediates mitochondrial autophagy and protects the heart against energy stress. Circ Res. (2015) 116:264–78. doi: 10.1161/circresaha.116.303356 25332205

[47] Jetto CT Nambiar A Manjithaya R. Mitophagy and neurodegeneration: between the knowns and the unknowns. Front Cell Dev Biol. (2022) 10. doi: 10.3389/fcell.2022.837337 PMC8981085 35392168

[48] Friedman JR Lackner LL West M DiBenedetto JR Nunnari J Voeltz GK. ER tubules mark sites of mitochondrial division. Science. (2011) 334:358–62. doi: 10.1126/science.1207385 PMC3366560 21885730

[49] Böckler S Westermann B. Mitochondrial ER contacts are crucial for mitophagy in yeast. Dev Cell. (2014) 28:450–8. doi: 10.1016/j.devcel.2014.01.012 24530295

[50] Böckler S Westermann B. ER-mitochondria contacts as sites of mitophagosome formation. Autophagy. (2014) 10:1346–7. doi: 10.4161/auto.28981 PMC4203561 24905224

[51] Garofalo T Matarrese P Manganelli V Marconi M Tinari A Gambardella L. Evidence for the involvement of lipid rafts localized at the ER-mitochondria associated membranes in autophagosome formation. Autophagy. (2016) 12:917–35. doi: 10.1080/15548627.2016.1160971 PMC4922444 27123544

[52] Ham SJ Yoo H Woo D Lee DH Park KS Chung J. PINK1 and Parkin regulate IP R-mediated ER calcium release. Nat Commun. (2023) 14(1):5202. doi: 10.1038/s41467-023-40929-z 37626046 PMC10457342

[53] Kuroda Y Mitsui T Kunishige M Shono M Akaike M Azuma H. Parkin enhances mitochondrial biogenesis in proliferating cells. Hum Mol Genet. (2006) 15:883–95. doi: 10.1093/hmg/ddl006 16449237

[54] Vincow ES Merrihew G Thomas RE Shulman NJ Beyer RP MacCoss MJ. The PINK1-Parkin pathway promotes both mitophagy and selective respiratory chain turnover *in vivo* . Proc Natl Acad Sci USA. (2013) 110:6400–5. doi: 10.1073/pnas.1221132110 PMC3631677 23509287

[55] Docherty CK Bresciani J Carswell A Chanderseka A Friel E Stasi M. An inducible and vascular smooth muscle cell-specific pink1 knockout induces mitochondrial energetic dysfunction during atherogenesis. Int J Mol Sci. (2021) 22:9993. doi: 10.3390/ijms22189993 34576157 PMC8467198

[56] Gelmetti V De Rosa P Torosantucci L Marini ES Romagnoli A Di Rienzo M. PINK1 and BECN1 relocalize at mitochondria-associated membranes during mitophagy and promote ER-mitochondria tethering and autophagosome formation. Autophagy. (2017) 13:654–69. doi: 10.1080/15548627.2016.1277309 PMC5388214 28368777

[57] Yang J Sun M Cheng R Tan H Liu C Chen R. Pitavastatin activates mitophagy to protect EPC proliferation through a calcium-dependent CAMK1-PINK1 pathway in atherosclerotic mice. Commun Biol. (2022) 5:124. doi: 10.1038/s42003-022-03081-w 35145192 PMC8831604

[58] Zhang YK Wang S Chen X Wang Z Wang X Zhou Q. Liraglutide prevents high glucose induced HUVECs dysfunction via inhibition of PINK1/Parkin-dependent mitophagy. Mol Cell Endocrinol. (2022) 545:111560. doi: 10.1016/j.mce.2022.111560 35032624

[59] Chen X Mi L Gu G Gao X Gao X Shi M. Dysfunctional endoplasmic reticulum-mitochondrion coupling is associated with endoplasmic reticulum stress-induced apoptosis and neurological deficits in a rodent model of severe head injury. J Neurotrauma. (2022) 39:560–76. doi: 10.1089/neu.2021.0347 35018820

[60] Marchi S Patergnani S Missiroli S Morciano G Rimessi A Wieckowski MR. Mitochondrial and endoplasmic reticulum calcium homeostasis and cell death. Cell Calcium. (2018) 69:62–72. doi: 10.1016/j.ceca.2017.05.003 28515000

[61] Danese A Patergnani S Bonora M Wieckowski MR Previati M Giorgi C. Calcium regulates cell death in cancer: Roles of the mitochondria and mitochondria-associated membranes (MAMs). Biochim Et Biophys Acta-Bioenergetics. (2017) 1858:615–27. doi: 10.1016/j.bbabio.2017.01.003 28087257

[62] Yu SJ Zhang L Liu C Yang J Zhang J Huang L. PACS2 is required for ox-LDL-induced endothelial cell apoptosis by regulating mitochondria-associated ER membrane formation and mitochondrial Ca elevation. Exp Cell Res. (2019) 379:191–202. doi: 10.1016/j.yexcr.2019.04.002 30970236

[63] Chen WY Chen YF Chan HC Chung CH Peng HY Ho YC. Role of apolipoprotein E in electronegative low -density lipoprotein-induced mitochondrial dysfunction in cardiomyocytes. Metabolism-Clinical Exp. (2020) 107:154227. doi: 10.1016/j.metabol.2020.154227 32275974

[64] Zhang Y Li JJ Xu R Wang XP Zhao XY Fang Y. Nogo-B mediates endothelial oxidative stress and inflammation to promote coronary atherosclerosis in pressure-overloaded mouse hearts. Redox Biol. (2023) 68:102944. doi: 10.1016/j.redox.2023.102944 37890359 PMC10633694

[65] Baik SH Ramanujan VK Becker C Fett S Underhill DM Wolf AJ. Hexokinase dissociation from mitochondria promotes oligomerization of VDAC that facilitates NLRP3 inflammasome assembly and activation. Sci Immunol. (2023) 8:eade7652. doi: 10.1126/sciimmunol.ade7652 37327321 PMC10360408

[66] Dong YZ Lee Y Cui K He M Wang B Bhattacharjee S. Epsin-mediated degradation of IP3R1 fuels atherosclerosis. Nat Commun. (2020) 11:928. doi: 10.1038/s41467-020-17848-4 32770009 PMC7414107

[67] Santos NC Girik V Nunes-Hasler P. ORP5 and ORP8: sterol sensors and phospholipid transfer proteins at membrane contact sites? Biomolecules. (2020) 10. doi: 10.3390/biom10060928 PMC7356933 32570981

[68] Jaworski CJ Moreira E Li A Lee R Rodriguez IR. A family of 12 human genes containing oxysterol-binding domains. Genomics. (2001) 78:185–96. doi: 10.1006/geno.2001.6663 11735225

[69] Du X Kumar J Ferguson C Schulz TA Ong YS Hong W. A role for oxysterol-binding protein-related protein 5 in endosomal cholesterol trafficking. J Cell Biol. (2011) 192:121–35. doi: 10.1083/jcb.201004142 PMC3019559 21220512

[70] Galmes R Houcine A van Vliet AR Agostinis P Jackson CL Giordano F. ORP5/ORP8 localize to endoplasmic reticulum-mitochondria contacts and are involved in mitochondrial function. EMBO Rep. (2016) 17:800–10. doi: 10.15252/embr.201541108 PMC5278607 27113756

[71] Guyard V Monteiro-Cardoso VF Omrane M Sauvanet C Houcine A Boulogne C. ORP5 and ORP8 orchestrate lipid droplet biogenesis and maintenance at ER-mitochondria contact sites. J Cell Biol. (2022) 221:e202112107. doi: 10.1083/jcb.202112107 35969857 PMC9375143

[72] Pu MM Zheng W Zhang H Wan W Peng C Chen X. ORP8 acts as a lipophagy receptor to mediate lipid droplet turnover. Protein Cell. (2023) 14:653–67. doi: 10.1093/procel/pwac063 PMC10501187 37707322

[73] Eisenreichova A Klima M Anila MM Koukalova A Humpolickova J Różycki B. Crystal structure of the ORP8 lipid transport ORD domain: model of lipid transport. Cells. (2023) 12:1974. doi: 10.3390/cells12151974 37566053 PMC10417380

[74] Assis LHP Dorighello GdG Rentz T de Souza JC Vercesi AE de Oliveira HCF. *In vivo* pravastatin treatment reverses hypercholesterolemia induced mitochondria-associated membranes contact sites, foam cell formation, and phagocytosis in macrophages. Front Mol Biosci. (2022) 9:839428. doi: 10.3389/fmolb.2022.839428 35372506 PMC8965079

[75] Ding YQ Liu N Zhang D Guo L Shang Q Liu Y. Mitochondria-associated endoplasmic reticulum membranes as a therapeutic target for cardiovascular diseases. Front Pharmacol. (2024) 15:1398381. doi: 10.3389/fphar.2024.1398381 38694924 PMC11061472

[76] Li JY Xin Y Li J Chen H Li H. Phosphatidylethanolamine N-methyltransferase: from functions to diseases. Aging Dis. (2023) 14:879–91. doi: 10.14336/ad.2022.1025 PMC10187709 37191416

[77] Chandhok G Lazarou M Neumann B. Structure, function, and regulation of mitofusin-2 in health and disease. Biol Rev. (2018) 93:933–49. doi: 10.1111/brv.12378 PMC6446723 29068134

[78] Hu Y Chen H Zhang L Lin X Li X Zhuang H. The AMPK-MFN2 axis regulates MAM dynamics and autophagy induced by energy stresses. Autophagy. (2021) 17:1142–56. doi: 10.1080/15548627.2020.1749490 PMC8143230 32249716

[79] Jiao YY Zhang X Yang H Ma H Zou J. Mesoporous tantalum oxide nanomaterials induced cardiovascular endothelial cell apoptosis via mitochondrial-endoplasmic reticulum stress apoptotic pathway. Drug Delivery. (2023) 30:108–20. doi: 10.1080/10717544.2022.2147251 PMC9788694 36533874

[80] Feng J Lü S Ding Y Zheng M Wang X. Homocysteine activates T cells by enhancing endoplasmic reticulum-mitochondria coupling and increasing mitochondrial respiration. Protein Cell. (2016) 7:391–402. doi: 10.1007/s13238-016-0245-x 26856873 PMC4887324

[81] Dabravolski SA Sadykhov NK Kartuesov AG Borisov EE Sukhorukov VN Orekhov AN. Interplay between zn homeostasis and mitochondrial functions in cardiovascular diseases and heart ageing. Int J Mol Sci. (2022) 23:6890. doi: 10.3390/ijms23136890 35805904 PMC9266371

[82] Xu L Hao H Hao Y Wei G Li G Ma P. Aberrant MFN2 transcription facilitates homocysteine-induced VSMCs proliferation via the increased binding of c-Myc to DNMT1 in atherosclerosis. J Cell Mol Med. (2019) 23:4611–26. doi: 10.1111/jcmm.14341 PMC6584594 31104361

[83] Zhao JH Li J Li G Chen M. The role of mitochondria-associated membranes mediated ROS on NLRP3 inflammasome in cardiovascular diseases. Front Cardiovasc Med. (2022) 9:1059576. doi: 10.3389/fcvm.2022.1059576 36588561 PMC9794868

[84] Xie H Tang J Song L Xu G Li W Zhu J. Mitochondria-endoplasmic reticulum crosstalk in apoptosis: The interactions of cytochrome c with monooxygenase and its reductase. Int J Biol Macromolecules. (2024) 279:135160. doi: 10.1016/j.ijbiomac.2024.135160 39214221

[85] Guo W Diao Z Liu W. Asymmetric dimethylarginine downregulates sarco/endoplasmic reticulum calcium−ATPase 3 and induces endoplasmic reticulum stress in human umbilical vein endothelial cells. Mol Med Rep. (2017) 16:7541–7. doi: 10.3892/mmr.2017.7529 PMC5865888 28944875

[86] Mohammadi A Gholamhoseyniannajar A Yaghoobi MM Jahani Y Vahabzadeh Z. Expression levels of heat shock protein 60 and glucose-regulated protein 78 in response to trimethylamine-N-oxide treatment in murine macrophage J774A.1 cell line. Cell Mol Biol. (2015) 61:94–100. 26429299

[87] Moulis M Grousset E Faccini J Richetin K Thomas G Vindis C. The multifunctional sorting protein PACS-2 controls mitophagosome formation in human vascular smooth muscle cells through mitochondria-ER contact sites. Cells. (2019) 8:638. doi: 10.3390/cells8060638 31242668 PMC6627983

[88] Lin W Chen S Wang Y Wang M Lee WY Jiang X. Dynamic regulation of mitochondrial-endoplasmic reticulum crosstalk during stem cell homeostasis and aging. Cell Death Dis. (2021) 12:794. doi: 10.1038/s41419-021-03912-4 34400615 PMC8368094

[89] Larrea D Pera M Gonnelli A Quintana-Cabrera R Akman HO Guardia-Laguarta C. MFN2 mutations in Charcot-Marie-Tooth disease alter mitochondria-associated ER membrane function but do not impair bioenergetics. Hum Mol Genet. (2019) 28:1782–800. doi: 10.1093/hmg/ddz008 PMC6522073 30649465

[90] Granatiero V Giorgio V Calì T Patron M Brini M Bernardi P. Reduced mitochondrial Ca(2+) transients stimulate autophagy in human fibroblasts carrying the 13514A>G mutation of the ND5 subunit of NADH dehydrogenase. Cell Death Differ. (2016) 23:231–41. doi: 10.1038/cdd.2015.84 PMC4716301 26206091

[91] Ma Z Du X Sun Y Jia Y Liang X Gao Y. Attenuation of PM2.5-induced lung injury by 4-phenylbutyric acid: maintenance of [Ca(2+)]i stability between endoplasmic reticulum and mitochondria. Biomolecules. (2024) 14:1135. doi: 10.3390/biom14091135 39334901 PMC11430257

[92] Zhu GL Gao H Li Y Li X Yang X Wang C. Suppression of endoplasmic reticulum stress by 4-PBA enhanced atherosclerotic plaque stability via up-regulating CLOCK expression. Pathol Res Pract. (2024) 253:154969. doi: 10.1016/j.prp.2023.154969 38029715

[93] Laksono RM Kalim H Rohman MS Widodo N Ahmad MR Halim W. Pulsed radiofrequency decreases pERK and affects intracellular Ca^2+^ influx, cytosolic ATP level, and mitochondrial membrane potential in the sensitized dorsal root ganglion neuron induced by N-methyl D-aspartate. J Pain Res. (2023) 16:1697–711. doi: 10.2147/JPR.S425900 PMC10216856 37252110

[94] Shoshan-Barmatz V Anand U Nahon-Crystal E Di Carlo M Shteinfer-Kuzmine A. Adverse effects of metformin from diabetes to COVID-19, cancer, neurodegenerative diseases, and aging: is VDAC1 a common target? Front Physiol. (2021) 12:730048. doi: 10.3389/fphys.2021.730048 34671273 PMC8521008

[95] Jenkins AJ Welsh P Petrie JR. Metformin, lipids and atherosclerosis prevention. Curr Opin Lipidology. (2018) 29:346–53. doi: 10.1097/mol.0000000000000532 29878903

[96] Zhou WP Fan XR Li SH Zeng ZL Wei YM. Statins combined with AAV8-TBG-LOX-1 reduce the vascular lipid-driven inflammatory response and inhibit atherosclerosis. Curr Med Sci. (2024) 44:1097–102. doi: 10.1007/s11596-024-2954-3 39627476

[97] Yin Y Wu C Zhou Y Zhang M Mai S Chen M. Ezetimibe induces paraptosis through niemann-pick C1-like 1 inhibition of mammalian-target-of-rapamycin signaling in hepatocellular carcinoma cells. Genes (Basel). (2023) 15:4. doi: 10.3390/genes15010004 38275586 PMC10815321

[98] Ali AH Younis N Abdallah R Shaer F Dakroub A Ayoub MA. Lipid-lowering therapies for atherosclerosis: statins, fibrates, ezetimibe and PCSK9 monoclonal antibodies. Curr Med Chem. (2021) 28:7427–45. doi: 10.2174/1875533xmte03ndeo0 33655822

[99] Kong DZ Sun P Lu Y Yang Y Min DY Zheng SC. Yi Mai granule improve energy supply of endothelial cells in atherosclerosis via miRNA-125a-5p regulating mitochondrial autophagy through Pink1-Mfn2-Parkin pathway. J Ethnopharmacology. (2024) 319:117114. doi: 10.1016/j.jep.2023.117114 37678420

[100] Li ZY Wang B Tian L Zheng B Zhao X Liu R. Methane-rich saline suppresses ER-mitochondria contact and activation of the NLRP3 inflammasome by regulating the PERK signaling pathway to ameliorate intestinal ischemia-reperfusion injury. Inflammation. (2024) 47:376–89. doi: 10.1007/s10753-023-01916-0 PMC10799159 37898993

[101] Hong KX Wang J Kang XXue H Gao Y Liang H. Ferulic acid and protocatechuic acid alleviate atherosclerosis by promoting UCP1 expression to inhibit the NLRP3-IL-1β signaling pathway. Food Funct. (2025) 16:40–53. doi: 10.1039/d4fo02955k 39584934

[102] Chen W Zhang Y Miao G Ying Y Ren Z Sun X. The augment effects of magnesium hydride on the lipid lowering effect of atorvastatin: an *in vivo* and *in vitro* investigation. Med Gas Res. (2025) 15:148–55. doi: 10.4103/mgr.medgasres-d-23-00047 PMC11515074 39436189

[103] Aldossary KM Ali LS Abdallah MSBahaa MM Elmasry TA Elberri EI. Effect of a high dose atorvastatin as added-on therapy on symptoms and serum AMPK/NLRP3 inflammasome and IL-6/STAT3 axes in patients with major depressive disorder: randomized controlled clinical study. Front Pharmacol. (2024) 15:1464358. doi: 10.3389/fphar.2024.1381523 39268472 PMC11391526

[104] Li FY Xu D Hou K Gou X Lv N Fang W. Pretreatment of indobufen and aspirin and their combinations with clopidogrel or ticagrelor alleviates inflammasome mediated pyroptosis via inhibiting NF-κB/NLRP3 pathway in ischemic stroke. J Neuroimmune Pharmacol. (2021) 16:835–53. doi: 10.1007/s11481-020-09978-9 33512659

[105] Golledge J. Update on the pathophysiology and medical treatment of peripheral artery disease. Nat Rev Cardiol. (2022) 19:456–74. doi: 10.1038/s41569-021-00663-9 34997200

[106] Gutierrez T Parra V Troncoso R Pennanen C Contreras-Ferrat A Vasquez-Trincado C. Alteration in mitochondrial Ca(2+) uptake disrupts insulin signaling in hypertrophic cardiomyocytes. Cell Commun Signal. (2014) 12:68. doi: 10.1186/s12964-014-0068-4 25376904 PMC4234850

[107] Ruan H Huang Q Wan B Yang M. Curcumin alleviates lipopolysaccharides-induced inflammation and apoptosis in vascular smooth muscle cells via inhibition of the NF-kappaB and JNK signaling pathways. Inflammopharmacology. (2022) 30:517–25. doi: 10.1007/s10787-021-00912-w 35229255

[108] Li X Zhu J Lin Q Yu M Lu J Feng J. Effects of curcumin on mitochondrial function, endoplasmic reticulum stress, and mitochondria-associated endoplasmic reticulum membranes in the jejunum of oxidative stress piglets. J Agric Food Chem. (2022) 70:8974–85. doi: 10.1021/acs.jafc.2c02824 35849777

[109] Lee DH Kim HH Cho HJ Yu YB Kang HC Kim JL. Cordycepin-Enriched WIB801C from Cordyceps militaris Inhibits Collagen-Induced [Ca(2+)]i Mobilization via cAMP-Dependent Phosphorylation of Inositol 1, 4, 5-Trisphosphate Receptor in Human Platelets. Biomol Ther (Seoul). (2014) 22:223–31. doi: 10.4062/biomolther.2014.025 PMC4060073 25009703

[110] Gao J Wang H Li Y Li W. Resveratrol attenuates cerebral ischaemia reperfusion injury via modulating mitochondrial dynamics homeostasis and activating AMPK-Mfn1 pathway. Int J Exp Pathol. (2019) 100:337–49. doi: 10.1111/iep.12336 PMC7042761 31867811

[111] Zhang RF Jiang Y Zhang G Zeng W Suo Y Zhang F. Mitochondrial DNA in atherosclerosis: Mechanisms, biomarker potential, and therapeutic perspectives. Int Immunopharmacol. (2025) 152:114449. doi: 10.1016/j.intimp.2025.114449 40073813

[112] Kalia R Wang RY Yusuf A Thomas PV Agard DA Shaw JM. Structural basis of mitochondrial receptor binding and constriction by DRP1. Nature. (2018) 558:401–5. doi: 10.1038/s41586-018-0211-2 PMC6120343 29899447

[113] Liu Y Huang Y Xu C An P Luo Y Jiao L. Mitochondrial dysfunction and therapeutic perspectives in cardiovascular diseases. Int J Mol Sci. (2022) 23:16053. doi: 10.3390/ijms232416053 36555691 PMC9788331

[114] Longchamps RJ Castellani CA Yang SY Newcomb CE Sumpter JA Lane J. Evaluation of mitochondrial DNA copy number estimation techniques. PLoS One. (2020) 15:e0228166. doi: 10.1371/journal.pone.0228166 32004343 PMC6994099

[115] Cutlip DE Chhabra AG Baim DS Chauhan MS Marulkar S Massaro J. Beyond restenosis: five-year clinical outcomes from second-generation coronary stent trials. Circulation. (2004) 110:1226–30. doi: 10.1161/01.CIR.0000140721.27004.4B 15337693

[116] Hara T Rai Y. Carotid endarterectomy. Adv Tech Stand Neurosurg. (2022) 44:187–207. doi: 10.1007/978-3-030-87649-4_10 35107680

[117] Chen PP Patel PB Ding J Krimbill J Siracuse JJ O'Donnell TFX. Asian race is associated with peripheral arterial disease severity and postoperative outcomes. J Vasc Surg. (2023) 78:175–83.e3. doi: 10.1016/j.jvs.2023.02.015 36889608

[118] Arasaki K Shimizu H Mogari H Nishida N Hirota N Furuno A. A role for the ancient SNARE syntaxin 17 in regulating mitochondrial division. Dev Cell. (2015) 32:304–17. doi: 10.1016/j.devcel.2014.12.011 25619926

[119] Berger KH Sogo LF Yaffe MP. Mdm12p, a component required for mitochondrial inheritance that is conserved between budding and fission yeast. J Cell Biol. (1997) 136:545–53. doi: 10.1083/jcb.136.3.545 PMC2134291 9024686

[120] Esposito M Hermann-Le Denmat S Delahodde A. Contribution of ERMES subunits to mature peroxisome abundance. PLoS One. (2019) 14:e0214287. doi: 10.1371/journal.pone.0214287 30908556 PMC6433259

[121] Obara CJ Nixon-Abell J Moore AS Riccio F Hoffman DP Shtengel G. Motion of VAPB molecules reveals ER-mitochondria contact site subdomains. Nature. (2024) 626:169–76. doi: 10.1038/s41586-023-06956-y PMC10830423 38267577

[122] Simmen T Aslan JE Blagoveshchenskaya AD Thomas L Wa. PACS-2 controls endoplasmic reticulum-mitochondria communication and Bid-mediated apoptosis. EMBO J. (2005) 24:717–29. doi: 10.1038/sj.emboj.7600637 PMC549619 15692567

[123] Çoku J Booth DM Skoda JPedrotty MC Vogel J Liu K. Reduced ER-mitochondria connectivity promotes neuroblastoma multidrug resistance. EMBO J. (2022) 41:e108272. doi: 10.15252/embj.2021108272 35211994 PMC9016347

[124] Giorgi C Baldassari F Bononi ABonora M De Marchi E Marchi S. Mitochondrial ca(2+) and apoptosis. Cell Calcium. (2012) 52:36–43. doi: 10.1016/j.ceca.2012.02.008 22480931 PMC3396846

[125] Carpio MA Means RE Brill AL Sainz A Ehrlich BE Katz SG. BOK controls apoptosis by Ca transfer through ER-mitochondrial contact sites. Cell Rep. (2021) 34:108827. doi: 10.1016/j.celrep.2021.108827 33691099 PMC7995216

[126] Liu WK Song H Xu J Guo Y Zhang C Yao Y. Low shear stress inhibits endothelial mitophagy via caveolin-1/miR-7-5p/SQSTM1 signaling pathway. Atherosclerosis. (2022) 356:9–17. doi: 10.1016/j.atherosclerosis.2022.07.014 35952464

[127] Hamasaki M Furuta N Matsuda A Nezu A Yamamoto A Fujita N. Autophagosomes form at ER-mitochondria contact sites. Nature. (2013) 495:389–93. doi: 10.1038/nature11910 23455425

[128] Cao YH Chen X Pan FWang M Zhuang H Chen J. Xinmaikang-mediated mitophagy attenuates atherosclerosis via the PINK1/Parkin signaling pathway. Phytomedicine. (2023) 119:154955. doi: 10.1016/j.phymed.2023.154955 37572567

[129] Yao RQ Ren C Xia ZF Yao YM. Organelle-specific autophagy in inflammatory diseases: a potential therapeutic target underlying the quality control of multiple organelles. Autophagy. (2021) 17:385–401. doi: 10.1080/15548627.2020.1725377 32048886 PMC8007140

[130] Mao K Klionsky DJ. Mitochondrial fission facilitates mitophagy in. Autophagy. (2013) 9:1900–1. doi: 10.4161/auto.25804 PMC4028339 24025250

[131] Kohler V Aufschnaiter A Büttner S. Closing the gap: membrane contact sites in the regulation of autophagy. Cells. (2020) 9:1184. doi: 10.3390/cells9051184 32397538 PMC7290522

[132] Geisler S Holmström KM Skujat D Fiesel FC Rothfuss OC Kahle PJ. PINK1/Parkin-mediated mitophagy is dependent on VDAC1 and p62/SQSTM1. Nat Cell Biol. (2010) 12:119–31. doi: 10.1038/ncb2012 20098416

[133] El Kodsi DN Tokarew JM Sengupta R Lengacher NA Chatterji A Nguyen AP. Parkin coregulates glutathione metabolism in adult mammalian brain. Acta Neuropathol Commun. (2023) 11:19. doi: 10.1186/s40478-022-01488-4 36691076 PMC9869535

[134] Choi SH Agatisa-Boyle C Gonen A Kim A Kim J Alekseeva E. Intracellular AIBP (Apolipoprotein A-I binding protein) regulates oxidized LDL (Low-density lipoprotein)-induced mitophagy in macrophages. Arterioscler Thromb Vasc Biol. (2021) 41:E82–96. doi: 10.1161/atvbaha.120.315485 PMC8105271 33356389

[135] Wang ZX Sun W Zhang K Ke X Wang Z. New insights into the relationship of mitochondrial metabolism and atherosclerosis. Cell Signalling. (2025) 127:111580. doi: 10.1016/j.cellsig.2024.111580 39732307

[136] Zhng ZB Valero RA Quintana A Hoth M Núñez L Villalobos C. Research progress on the role of endoplasmic reticulum-mitochondrial interaction in cardiovascular diseases. Heart J. (2020) 32:522–7. doi: 10.1074/jbc.M110.198952

[137] Sreekumar PG Hinton DR Kannan R. Endoplasmic reticulum-mitochondrial crosstalk: a novel role for the mitochondrial peptide humanin. Neural Regeneration Res. (2017) 12:35–8. doi: 10.4103/1673-5374.198970 PMC5319229 28250736

[138] Yang YS Liu Y Wang Y Chao Y Zhang J Jia Y. Regulation of SIRT1 and its roles in inflammation. Front Immunol. (2022) 13:831168. doi: 10.3389/fimmu.2022.831168 35359990 PMC8962665

[139] Hall AR Burke N Dongworth RK Kalkhoran SB Dyson A Vicencio JM. Hearts deficient in both Mfn1 and Mfn2 are protected against acute myocardial infarction. Cell Death Dis. (2016) 7(5):e2238. doi: 10.1038/cddis.2016.139 27228353 PMC4917668

